# Supplementary material for: The role of CDK8 in mesenchymal stem cells in controlling osteoclastogenesis and bone homeostasis
Source: Stem Cell Reports. 2022 Jun 30;17(7):1576–88. doi: 10.1016/j.stemcr.2022.06.001 (PMC9287674; doi:10.1016/j.stemcr.2022.06.001)
Supplement: Document S1. Tables S1–S3 and Figures S1–S3 [file mmc1.pdf]

Stem Cell Reports, Volume *17*

Supplemental Information

The role of CDK8 in mesenchymal stem cells in controlling osteoclastogenesis and bone homeostasis

Takanori Yamada, Kazuya Fukasawa, Tetsuhiro Horie, Takuya Kadota, Jiajun Lyu, Kazuya Tokumura, Shinsuke Ochiai, Sayuki Iwahashi, Akane Suzuki, Gyujin Park, Rie Ueda, Megumi Yamamoto, Tatsuya Kitao, Hiroaki Shirahase, Hiroki Ochi, Shingo Sato, Takashi Iezaki, and Eiichi Hinoi

Supplementary Table 1. List of primers used for genotyping

Gene	Forward (5’-3’)	Reverse (5’-3’)
Cre	GAACCTGATGGACATGTTCAGG	AGTGCGTTCGAACGCTAGAGCCTGT
CDK8	CGTAGGTAGCAATCTGGTCGGGGT	CAGGTACACAGGCTGGATTTGCAC

Supplementary Table 2. List of primers used for deletion PCR.

Gene	Forward (5’-3’)	Reverse (5’-3’)
CDK8	CTTCCCTCTTCCCAGAGGAC	CAACCCCTTTTGAGGTTGAA

Supplementary Table 3. List of primers used for real-time PCR.

Gene	Forward (5’-3’)	Reverse (5’-3’)
Acp5	AGCTTCTCTGCCCTGGTACTC	TGGCTGTGGGATCAGTTGGTG
Cdkn1a	TCGCTGTCTTGCACTCTGGTGT	CCAATCTGCGCTTGGAGTGATAG
Ctsk	CAACTCTACTTCCCTTCTCTCTGC	GAGGACTCCAATGTCTACCAGC
Gapdh	AGGTCGGTGTGAACGGATTTG	TGTAGACCATGTAGTTGAGGTCA
Icam1	TTCACACTGAATGCCAGCTC	GTCTGCTGAGACCCCTCTTG
Ifi204	GGAAAGAGACAACCAAGAGC	TGGCTTGTAGTTGATGTAGG
Irf1	TCCAAGTCCAGCCGAGACACTA	ACTGCTGTGGTCATCAGGTAGG
Irf8	TCCAAGTCCAGCCGAGACAC	ACTGCTGTGGTCATCAGGTA
Mmp9	TGTGCGTTATGGTTCAGGTCAG	CTGCCAGGAAGACACTTGGTTATC
Smad7	AAGATCGGCTGTGGCATC	CCAACAGCGTCCTGGAGT
Spi1	ATCCAGAAGGGCAACCGCAAG	TCTTGACTTTCTTCACCTCGCCTG
Tap1	GACTCCTTGCTCTCCACTCA	AACGCTGTCACCGTTCCAGG
Tnfrsf11b	CTGCAATACACACACTCATCACT	ACCCAGAAACTGGTCATCAGC
Tnfsf11	TGTACTTTCGAGCGCAGATG	CCACAATGTGTTGCAGTTCC

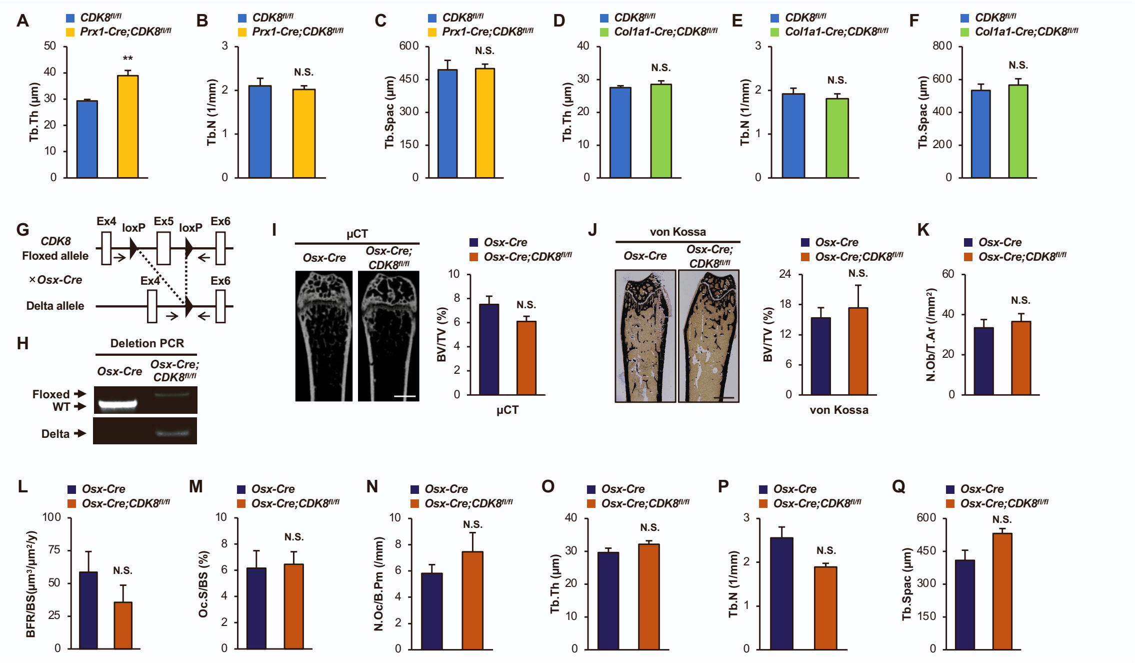

Supplementary Figure 1. Skeletal phenotype analysis of conditional MSC-specific, osteoblast-specific, and osteoprogenitor-specific *CDK8* knockout mice.

(A-C) Tb.Th (A), Tb.N (B) and Tb.Spac (C) measured by μCT of femurs from *CDK8*$^{fl/fl}$ and *Prx1-Cre;CDK8*$^{fl/fl}$ mice (n = 6-7, $^{**}P < 0.01$). (D-F) Tb.Th (D), Tb.N (E) and Tb.Spac (F) measured by μCT of femurs from *CDK8*$^{fl/fl}$ and *Col1a1-Cre;CDK8*$^{fl/fl}$ mice (n = 6-7). (G) Schematic diagram of generation of tissue-specific *CDK8* knockout mice. Black arrows represent primer binding sites. (H) Deletion efficiency of *CDK8* in the marrow-flushed bone of *Osx-Cre;CDK8*$^{fl/fl}$ mice at the genomic DNA level. (I) μCT analysis and BV/TV measurement of femurs from *Osx-Cre* and *Osx-Cre;CDK8*$^{fl/fl}$ mice (n = 4-5). (J to N) BV/TV determined by von Kossa staining (J), N.Ob/T.Ar (K), BFR/BS (L), Oc.S/BS (M) and N.Oc/B.Pm (N) of femur from *Osx-Cre* and *Osx-Cre;CDK8*$^{fl/fl}$ mice (n = 4-5). (O-Q) Tb.Th (O), Tb.N (P) and Tb.Spac (Q) measured by μCT of femurs from *CDK8*$^{fl/fl}$ and *Osx-Cre;CDK8*$^{fl/fl}$ mice (n = 4-5). All mice used in this study were female. Scale bars, 1 mm (I and J). μCT, micro-computed tomography; Tb.Th, Trabecular thickness; Tb.N, Trabecular number; Tb.Spac; Trabecular spacing; BV/TV, bone volume/tissue volume; N.Ob/T.Ar, number of osteoblasts/tissue area; BFR/BS, bone formation rate/bone surface; Oc.S/BS, osteoclast surface/bone surface; N.Oc/B.Pm, number of osteoclasts/bone perimeter; N.S., not significant.

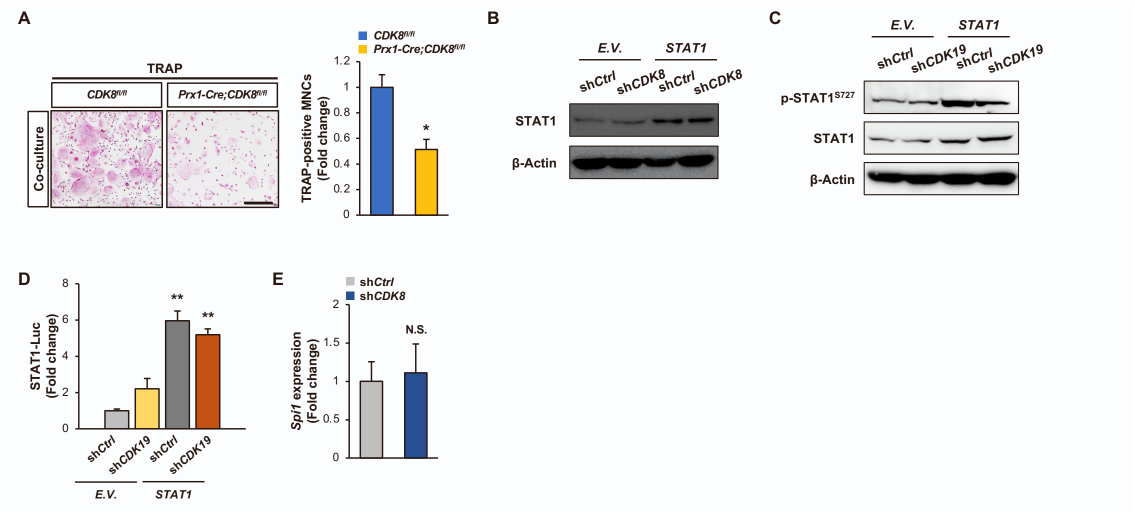

Supplementary Figure 2. Co-culture of MSCs from *Prx1-Cre;CDK8$^{fl/fl}$* mice and WT-BMMs, and expression profiles of *CDK8* and *CDK19* knockdown MSCs.

(A) BMMs prepared from WT mice were co-cultured with MSCs from *Prx1-Cre;CDK8$^{fl/fl}$* mice, followed by TRAP staining (n = 3 independent replicates, $^{*}P < 0.05$). (B) Protein levels of STAT1 in MSCs infected with sh*CDK8* in combination with *STAT1* expression vector. β-Actin served as a loading control. (C) Protein levels of STAT1 and p-STAT1^{Ser727} in MSCs infected with sh*CDK19* in combination with *STAT1* expression vector. β-Actin served as a loading control. (D) GAS-luc activity in MSCs infected with sh*CDK19* in combination with *STAT1* expression vector (n = 3 independent replicates, $^{**}P < 0.01$). (E) mRNA levels of *Spi1* in MSCs infected with sh*CDK8* (n = 4 independent replicate). Scale bar, 800 μm (A). BMM, bone marrow macrophage; MSC, mesenchymal stem cell; TRAP, tartrate-resistant acid phosphatase; sh*Ctrl*, sh*Control*; E.V., empty vector; N.S., not significant.

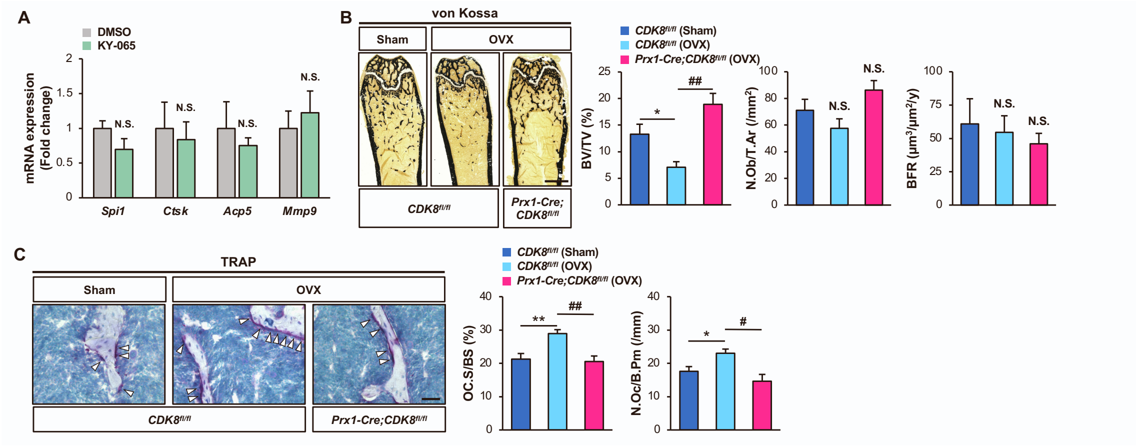

Supplementary Figure 3. Expression levels of osteoclastic marker genes in BMMs treated by KY-065, and the protective effect of MSC-specific *CDK8* deficiency on ovariectomy-induced bone loss.

(A) mRNA levels of *Spi1*, *Ctsk*, *Acp5* and *Mmp9* in MSCs treated with 30 nM KY-065 (n = 3 independent replicates). (B) BV/TV measured by von Kossa staining, N.Ob/T. Ar determined by toluidine blue staining and BFR/BS determined by calcein labeling of femurs from *CDK8*$^{fl/fl}$ and *Prx1-Cre;CDK8*$^{fl/fl}$ mice (n = 5-8, $^{*}P < 0.05$ (*CDK8*$^{fl/fl}$ (sham) vs *CDK8*$^{fl/fl}$ (OVX)), $^{\#\#}P < 0.01$ (*CDK8*$^{fl/fl}$ (OVX) vs *Prx1-Cre;CDK8*$^{fl/fl}$(OVX))). (C) Oc.S/BS and N.Oc/B.Pm determined by TRAP staining of femur (n = 5-8, $^{*}P < 0.05$ (*CDK8*$^{fl/fl}$ (sham) vs *CDK8*$^{fl/fl}$ (OVX)), $^{**}P < 0.01$ (*CDK8*$^{fl/fl}$ (sham) vs *CDK8*$^{fl/fl}$ (OVX)), $^{\#}P < 0.05$ (*CDK8*$^{fl/fl}$ (OVX) vs *Prx1-Cre;CDK8*$^{fl/fl}$(OVX)), $^{\#\#}P < 0.01$ (*CDK8*$^{fl/fl}$ (OVX) vs *Prx1-Cre;CDK8*$^{fl/fl}$(OVX))). Scale bars, 1 mm (B), 50 μm (C). OVX, ovariectomy; BV/TV, bone volume/tissue volume; N.Ob/T.Ar, number of osteoblasts/tissue area; BFR/BS, bone formation rate/bone surface; TRAP, tartrate-resistant acid phosphatase; Oc.S/BS, osteoclast surface/bone surface; N.Oc/B.Pm, number of osteoclasts/bone perimeter; N.S., not significant.
