# Supplementary material for: The role of CDK8 in mesenchymal stem cells in controlling osteoclastogenesis and bone homeostasis
Source: Stem Cell Reports. 2022 Jun 30;17(7):1576–88. doi: 10.1016/j.stemcr.2022.06.001 (PMC9287674; doi:10.1016/j.stemcr.2022.06.001)
Supplement: Document S2. Article plus supplemental information [file mmc2.pdf]

Stem Cell Reports
Article
OPEN ACCESS

The role of CDK8 in mesenchymal stem cells in controlling osteoclastogenesis and bone homeostasis

Takanori Yamada,[1,6] Kazuya Fukasawa,[1,6] Tetsuhiro Horie,[1,6] Takuya Kadota,[1,2] Jiajun Lyu,[1] Kazuya Tokumura,[1] Shinsuke Ochiai,[1] Sayuki Iwahashi,[1] Akane Suzuki,[1] Gyujin Park,[1] Rie Ueda,[1] Megumi Yamamoto,[2] Tatsuya Kitao,[2] Hiroaki Shirahase,[2] Hiroki Ochi,[3] Shingo Sato,[4] Takashi Iezaki,[1] and Eiichi Hinoi[1,5,*]

[1]Department of Bioactive Molecules, Pharmacology, Gifu Pharmaceutical University, Gifu 501-1196, Japan
[2]Drug Discovery Research Department, Kyoto Pharmaceutical Industries, Kyoto, Japan
[3]Department of Physiology and Cell Biology, Tokyo Medical and Dental University, Graduate School, Tokyo 113-8510, Japan
[4]Center for Innovative Cancer Treatment, Tokyo Medical and Dental University, Tokyo 113-8519, Japan
[5]United Graduate School of Drug Discovery and Medical Information Sciences, Gifu University, Gifu 501-1196, Japan
[6]These authors contributed equally
*Correspondence: hinoi-e@gifu-pu.ac.jp
https://doi.org/10.1016/j.stemcr.2022.06.001

SUMMARY

Bone marrow mesenchymal stem cells (MSCs) are critical regulators of postnatal bone homeostasis. Osteoporosis is characterized by bone volume and strength deterioration, partly due to MSC dysfunction. Cyclin-dependent kinase 8 (CDK8) belongs to the transcription-related CDK family. Here, CDK8 in MSCs was identified as important for bone homeostasis. *CDK8* level was increased in aged MSCs along with the association with aging-related signals. Mouse genetic studies revealed that CDK8 in MSCs plays a crucial role in bone resorption and homeostasis. Mechanistically, CDK8 in MSCs extrinsically controls osteoclastogenesis through the signal transducer and transcription 1 (STAT1)-receptor activator of the nuclear factor κ B ligand (RANKL) axis. Moreover, aged MSCs have high osteoclastogenesis-supporting activity, partly through a CDK8-dependent manner. Finally, pharmacological inhibition of CDK8 effectively repressed MSC-dependent osteoclastogenesis and prevented ovariectomy-induced osteoclastic activation and bone loss. These findings highlight that the CDK8-STAT1-RANKL axis in MSCs could play a crucial role in bone resorption and homeostasis.

INTRODUCTION

Osteoporosis leads to increased bone fragility and risk of bone fractures, which are often associated with morbidity, prolonged hospitalization, and increased public health burden (Cauley, 2013; Manolagas and Parfitt, 2010; Rachner et al., 2011). The incidence of osteoporosis has significantly increased due to a dramatic increase in the aging population worldwide (Curtis and Safford, 2012). Bone integrity and bone remodeling are coordinately regulated by two different types of cells—bone-forming osteoblasts and bone-resorbing osteoclasts in the bone marrow microenvironment (Harada and Rodan, 2003; Teitelbaum and Ross, 2003). Osteoblasts and osteoclasts are finely coupled to maintain proper bone mass, quality, and strength in the young or healthy bone marrow microenvironment, whereas their sophisticated regulation is imbalanced in the old or osteoporotic marrow microenvironment, resulting in osteoporosis pathogenesis and increased susceptibility to fracture (Feng and McDonald, 2011; Karsenty et al., 2009).

Bone marrow mesenchymal stem cells (MSCs), types of tissue-specific stem cells, are plastic adherent and multipotent cells that can be differentiated into osteoblasts, adipocytes, and chondrocytes *in vitro* (Kfoury and Scadden, 2015). MSCs are critical regulators of postnatal bone homeostasis, because they function as an osteoblast reservoir throughout the lifespan (Zhou et al., 2014). Aging gradually decreases the proliferation potential and function of MSCs, resulting in decreased osteoblast generation and leading to bone loss and reduced regenerative potential of the bone over time (Ambrosi et al., 2017; Zhou et al., 2008). MSCs from postmenopausal women with osteoporosis had a weaker osteogenic potential (Dalle Carbonare et al., 2009; Shen et al., 2020; You et al., 2016). In addition, an increased commitment of MSCs to adipogenic lineages by various factors, such as aging, menopause, and ovariectomy, also contributes to decreased osteoblast generation and bone loss (Ambrosi et al., 2017; Tokuzawa et al., 2010; Yu et al., 2018).

Cyclin-dependent kinases (CDKs) are a family of 20 serine/threonine kinases, which are broadly divided into two major subclasses: cell-cycle- and transcription-related CDKs (Lim and Kaldis, 2013). CDK8 belongs to the transcription-related CDKs, which comprise 5 subfamilies represented by CDK7, CDK8, CDK9, CDK12, and CDK13 and are involved in modulating transcriptional processes (Malumbres, 2014). Although chemical CDK8/19 kinase inhibitor has been reported to suppress osteoclastogenesis and promote osteoblastogenesis (Amirhosseini et al., 2019), the functional role and underlying mechanisms of CDK8 expressed in MSCs on bone homeostasis under

 | © 2022 The Author(s).
This is an open access article under the CC BY license (http://creativecommons.org/licenses/by/4.0/).

physiological and pathological conditions remain unknown both *in vitro* and *in vivo*.

Here, we found that the *CDK8* level was increased in aged MSCs, osteoprogenitors, and osteoblasts. Mouse genetic studies revealed that CDK8 played a crucial role in bone resorption and homeostasis through its expression in MSCs rather than in osteoprogenitors or osteoblasts. Subsequent analyses revealed that CDK8 in MSCs regulates non-cell-autonomous osteoclastogenesis through signal transducers and transcription 1 (STAT1)-receptor activator of nuclear factor κ-B (NF-κB) ligand (RANKL) axis. Furthermore, CDK8 in MSCs contributed to the age-associated increase in MSC-dependent non-cell-autonomous osteoclastogenesis. Finally, the pharmacological inhibition of CDK8 effectively repressed MSC-dependent osteoclastogenesis without affecting cell-autonomous osteoclastogenesis, preventing ovariectomy-induced osteoclastic activation and bone loss. Our findings demonstrated that the CDK8-STAT1-RANKL axis in MSCs could play a crucial role in maintaining bone resorption and homeostasis, indicating that targeting MSCs through CDK8 inhibition could be a promising strategy against various metabolic bone diseases associated with abnormal osteoclastogenesis, including age-related osteoporosis and postmenopausal osteoporosis.

RESULTS

Aging increases *CDK8* expression level in MSCs and their progeny

To investigate the *CDK8* level in MSCs and their progeny that contribute to skeletal homeostasis, a single-cell RNA sequencing (scRNA-seq) dataset deposited on the Gene Expression Omnibus (GEO) database was analyzed (Zhong et al., 2020). Five clusters were successfully identified: MSCs (cluster 1; *Prx1*$^+$, *Lepr*$^+$) and their descendants (osteoblast progenitors [cluster 2; *Hey1*$^+$, *Wif1*$^+$], osteoblasts [cluster 3; *Sp7*$^+$, *Col1a1*$^+$], osteocytes [cluster 4; *Dmp1*$^+$, *Bglap*$^+$] and chondrocytes [cluster 5; *Sox9*$^+$, *Acan*$^+$]) after removing hematopoietic, endothelial, adipogenic cells and pericytes through t-distributed stochastic neighbor embedding (t-SNE) cluster analysis (Figure 1A). *CDK8* expression level was significantly higher in aged MSCs, osteoprogenitors, and osteoblasts obtained from 16-month-old mice than that of young cells obtained from 1-, 1.5-, and 3-month-old mice; however, their levels were comparable among all age groups (1-, 1.5-, 3-, and 16-month-old mice) in osteocytes and chondrocytes (Figure 1B). In transcription-related CDKs, the expression levels of *CDK12*, *CDK13*, and *CDK19*, the paralog of *CDK8*, were also significantly higher in aged MSCs than in young MSCs, whereas the *CDK8* level was the highest among the transcription-related CDKs in MSCs (Figures 1C and 1D). A scRNA-seq data analysis also revealed that *CDK8* expression level was the highest in MSCs among 5 clusters (clusters 1–5) (Figure 1E). We confirmed that the CDK8 protein level was significantly higher in MSCs compared with that in osteoblasts and chondrocytes, along with significant higher phosphorylation levels of STAT1^{Ser727} and STAT3^{Ser727}, known direct targets of CDK8 (Bancerek et al., 2013; Martinez-Fabregas et al., 2020), in MSCs (Figure 1F). Moreover, a gene set enrichment analysis (GSEA) revealed that cell aging/cellular senescence-related pathway signature genes were significantly enriched in *CDK8*^{high} MSCs, *CDK8*^{high} osteoprogenitors, and *CDK8*^{high} osteoblasts (Figures 1G and 1H). In addition, a significantly higher *CDK8* expression level was confirmed in aged MSCs (PDGFRα$^+$ Sca1$^+$ cells) obtained from 2-year-old mice than in juvenile MSCs obtained from 2-week-old mice in a publicly available bulk RNA-seq dataset (Helbling et al., 2019) (Figure 1I). The CDK8 protein level was significantly higher in aged MSCs obtained from 16-month-old mice than that in young MSCs from 3-month-old mice (Figure 1J). Aged MSCs increased in size, but the CDK8 protein level was significantly increased in aged MSCs even though its level was normalized by cell size (Figure 1K).

These analyses suggest that CDK8 may have a possible association with skeletal aging through its expression in MSCs and their progeny (osteoprogenitors and osteoblasts).

CDK8 deletion in MSCs decreases osteoclastic activity, leading to high bone mass

To evaluate the importance of CDK8 in MSCs and their progeny on skeletal homeostasis *in vivo*, MSC-specific, osteoblast-specific, and osteoprogenitor-specific *CDK8*-knockout mice were generated by crossing *CDK8*-floxed mice with either *Paired-related homeobox 1* (*Prx1-Cre*), *Collagen type 1 alpha 1* (*Col1a1*)*-Cre* or *Osterix* (*Osx*)*-Cre* transgenic mice (Dacquin et al., 2002; Logan et al., 2002; Menzl et al., 2019; Rodda and McMahon, 2006) (Figures 2A and S1G). MSC-specific *CDK8*-knockout mice, called *Prx1-Cre;CDK8*$^{fl/fl}$ mice, showed no overt phenotypic abnormalities, such as the physical appearance, body weight, and naso-anal length (data not shown). A marked Cre-mediated excision was confirmed in the long bone of *Prx1-Cre;CDK8*$^{fl/fl}$ mice at the genomic DNA and mRNA levels (Figure 2B). *Prx1-Cre;CDK8*$^{fl/fl}$ mice displayed a significantly higher bone volume/tissue volume (BV/TV) and trabecular thickness (Tb.Th) in the femur than that of the control mice according to both micro-computed tomography (μCT) and histological analyses (Figures 2C, 2D, and S1A–S1C). Bone histomorphometric analyses revealed that bone formation indices, osteoblast number (N.Ob/T.Ar), and bone formation rate (BFR) were not more significantly changed in *Prx1-Cre;CDK8*$^{fl/fl}$ mice than that in the control mice (Figures 2E and 2F). Conversely, osteoclast

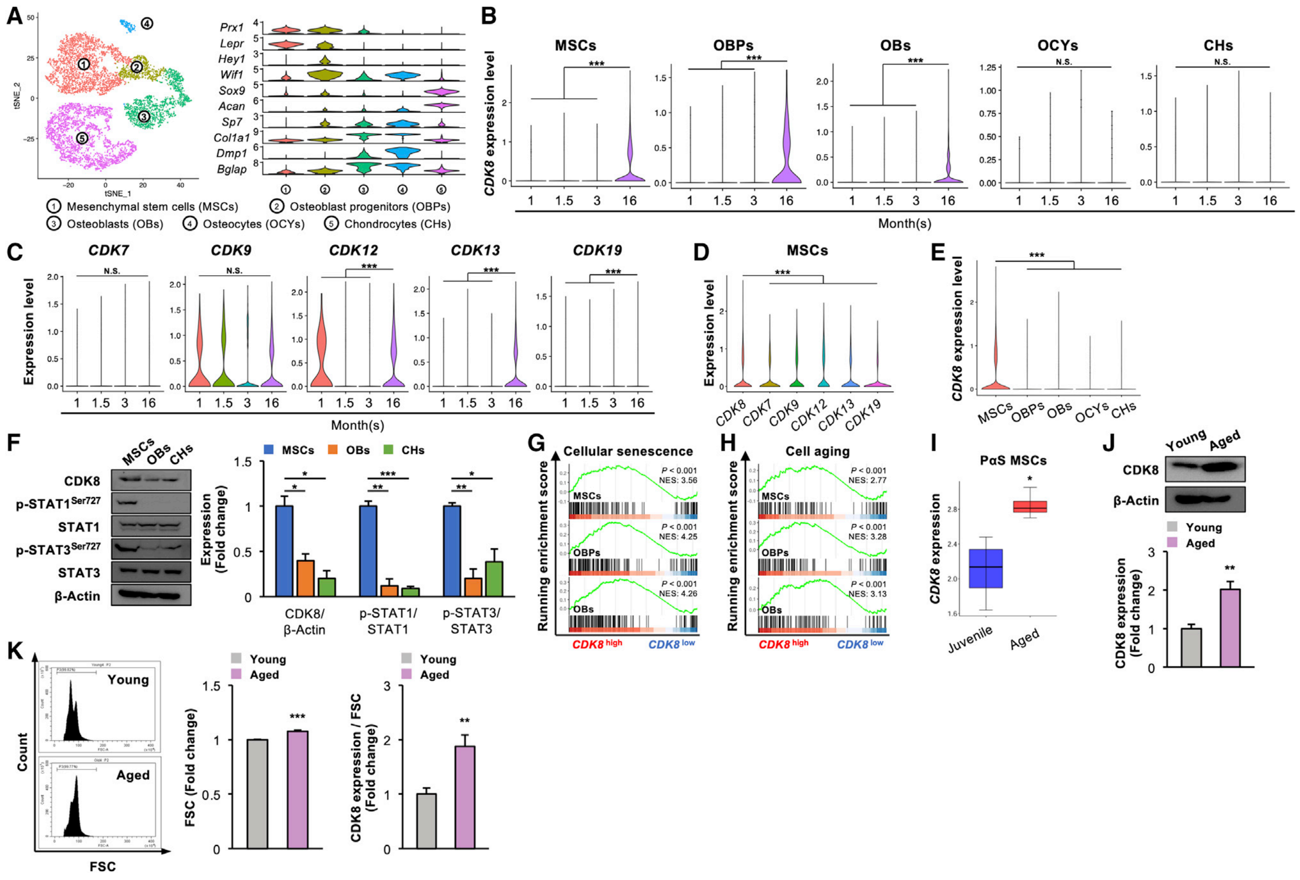

Figure 1. *CDK8* expression level is increased in MSCs and their progeny with aging

(A) The t-SNE plot of color-coded clustering of 1-, 1.5-, 3-, and 16-month cells (top, n = 8,753 cells) and the violin plots of cell-specific marker gene expression (bottom).

(B) *CDK8* expression levels of each cluster by age (***$p < 0.001$).

(C) Expression levels of transcription-related CDKs at the indicated time points of MSCs (***$p < 0.001$).

(D) Expression levels of transcription-related CDKs expressed in 1-, 1.5-, 3-, and 16-month MSCs (***$p < 0.001$).

(E) *CDK8* expression levels of 1-, 1.5-, 3-, and 16-month datasets among each cluster (***$p < 0.001$).

(F) Protein levels of CDK8, p-STAT1^{Ser727}, STAT1, p-STAT3^{Ser727} and STAT3 in MSCs, OBs, and CHs. β-Actin served as a loading control (n = 3 independent replicates, *$p < 0.05$, **$p < 0.01$, ***$p < 0.001$).

(G and H) The enrichment plots for aging-related gene sets in MSCs, OBPs, and OBs.

(I) *CDK8* expression levels in PαS MSCs compared with juvenile (2-week-old) and aged (2-year-old) mice (n = 4, *$p < 0.05$).

(J) Protein levels of CDK8 in aged MSCs. β-Actin served as a loading control (n = 4 independent replicates, **$p < 0.01$).

(K) Flow cytometric analysis of cell size in aged MSCs (n = 4 independent replicates, ***$p < 0.001$) and protein levels of CDK8 normalized by cell size (n = 4 independent replicates, **$p < 0.01$). Statistical significance was detected using the Steel test in (D).

t-SNE, t-distributed stochastic neighbor embedding; MSC, mesenchymal stem cell; OBP, osteoblast progenitor; OB, osteoblast; OCYs, osteocytes; CHs, chondrocytes; PαS, PDGFRα^{+} Sca-1^{+}; FSC, forward scatter.

surface (Oc.S/BS) and osteoclast number (N.Oc/B.Pm), bone resorption indices, were significantly lower in *Prx1-Cre;CDK8$^{fl/fl}$* mice than that in the control mice (Figure 2G), concomitant with a significant decreased circulating tartrate-resistant acid phosphatase (TRAP) level (Figure 2H). On the contrary, neither osteoblast-specific nor osteoprogenitor-specific *CDK8*-knockout mice, called *Col1a1-Cre;CDK8$^{fl/fl}$* and *Osx-Cre;CDK8$^{fl/fl}$* mice, respectively, showed significant changes in any bone phenotypes such as bone volume, trabecular parameters, and osteoblastic and osteoclastic parameters (Figures 2I–2O and S2D–S2Q).

Collectively, these results indicate that CDK8 may play a pivotal role in bone resorption and homeostasis through its expression in MSCs rather than osteoprogenitors or osteoblasts *in vivo*.

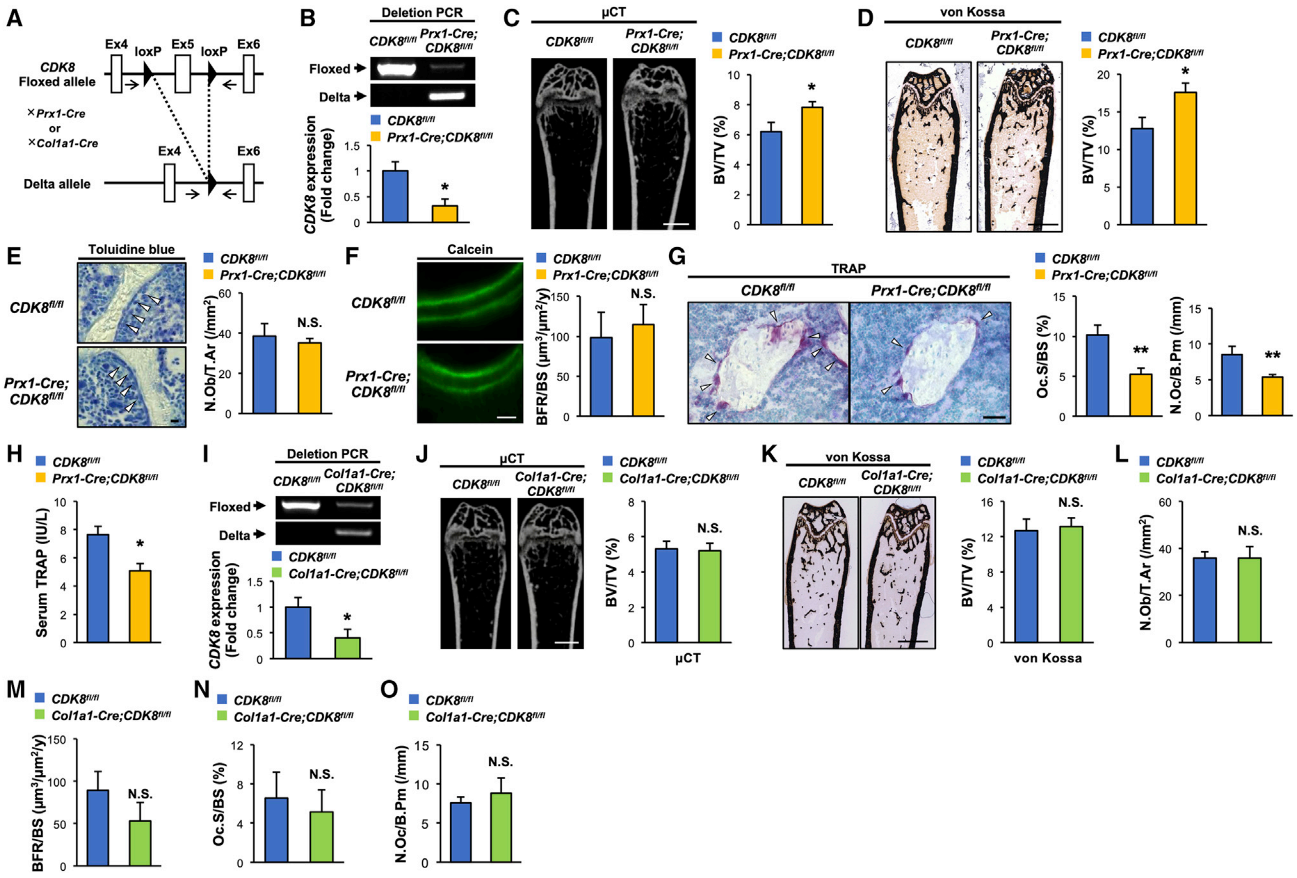

Figure 2. *CDK8* deficiency in MSCs suppresses osteoclastic activities, leading to high bone mass

(A) Schematic diagram of generation of tissue-specific *CDK8* knockout mice. Black arrows represent primer binding sites.

(B) Deletion efficiency of *CDK8* in the marrow-flushed bone of *Prx1-Cre;CDK8$^{fl/fl}$* at the genomic DNA and mRNA levels (n = 4 independent replicates, *p < 0.05).

(C and D) μCT (C) and von Kossa staining (D) and BV/TV measured by each method of femurs from *CDK8$^{fl/fl}$* and *Prx1-Cre;CDK8$^{fl/fl}$* mice (n = 6–7, *p < 0.05).

(E) Toluidine blue staining (left) and N.Ob/T.Ar of femurs from *CDK8$^{fl/fl}$* and *Prx1-Cre;CDK8$^{fl/fl}$* mice (n = 6–7). Arrowheads indicate osteoblasts.

(F) Calcein labeling (left) and BFR/BS of femurs from *CDK8$^{fl/fl}$* and *Prx1-Cre;CDK8$^{fl/fl}$* mice (n = 6–7).

(G) TRAP staining (left) and Oc.S/BS of femurs from *CDK8$^{fl/fl}$* and *Prx1-Cre;CDK8$^{fl/fl}$* mice (n = 6–7, **p < 0.01). Arrowheads indicate TRAP-positive osteoclasts.

(H) TRAP serum level in *CDK8$^{fl/fl}$* and *Prx1-Cre;CDK8$^{fl/fl}$* mice (n = 6–8, *p < 0.05).

(I) Deletion efficiency of *CDK8* in the marrow-flushed bone of *Col1a1-Cre;CDK8$^{fl/fl}$* at the genomic DNA level and the mRNA level (n = 4, *p < 0.05).

(J) μCT analysis and BV/TV measurement of femurs from *CDK8$^{fl/fl}$* and *Col1a1-Cre;CDK8$^{fl/fl}$* mice (n = 6–7).

(K–O) BV/TV measured by von Kossa staining (K), N.Ob/T.Ar (L), BFR/BS (M), Oc.S/BS (N), and N.Oc/B. Pm of femur from *CDK8$^{fl/fl}$* and *Col1a1-Cre;CDK8$^{fl/fl}$* mice (n = 6–7) (O).

All of the mice used in this study were female. Scale bars, 1 mm (C, D, J, and K), 50 μm (G), and 10 μm (E and F). μCT, micro-computed tomography; BV/TV, bone volume/tissue volume; N.Ob/T.Ar, number of osteoblasts/tissue area; BFR/BS, bone formation rate/bone surface; TRAP, tartrate-resistant acid phosphatase; Oc.S/BS, osteoclast surface/bone surface; N.Oc/B.Pm, number of osteoclasts/bone perimeter; N.S., not significant.

CDK8 deficiency in MSCs represses osteoclastogenesis via the downregulation of the STAT1-RANKL axis

Figure 2 shows that *CDK8* deficiency in MSCs leads to decreased bone resorption without affecting bone formation *in vivo*, and MSCs have been reported to also support osteoclastogenesis, as extensively demonstrated in osteoblasts and osteocytes (Mbalaviele et al., 1999). Therefore, co-culture experiments were subsequently performed to

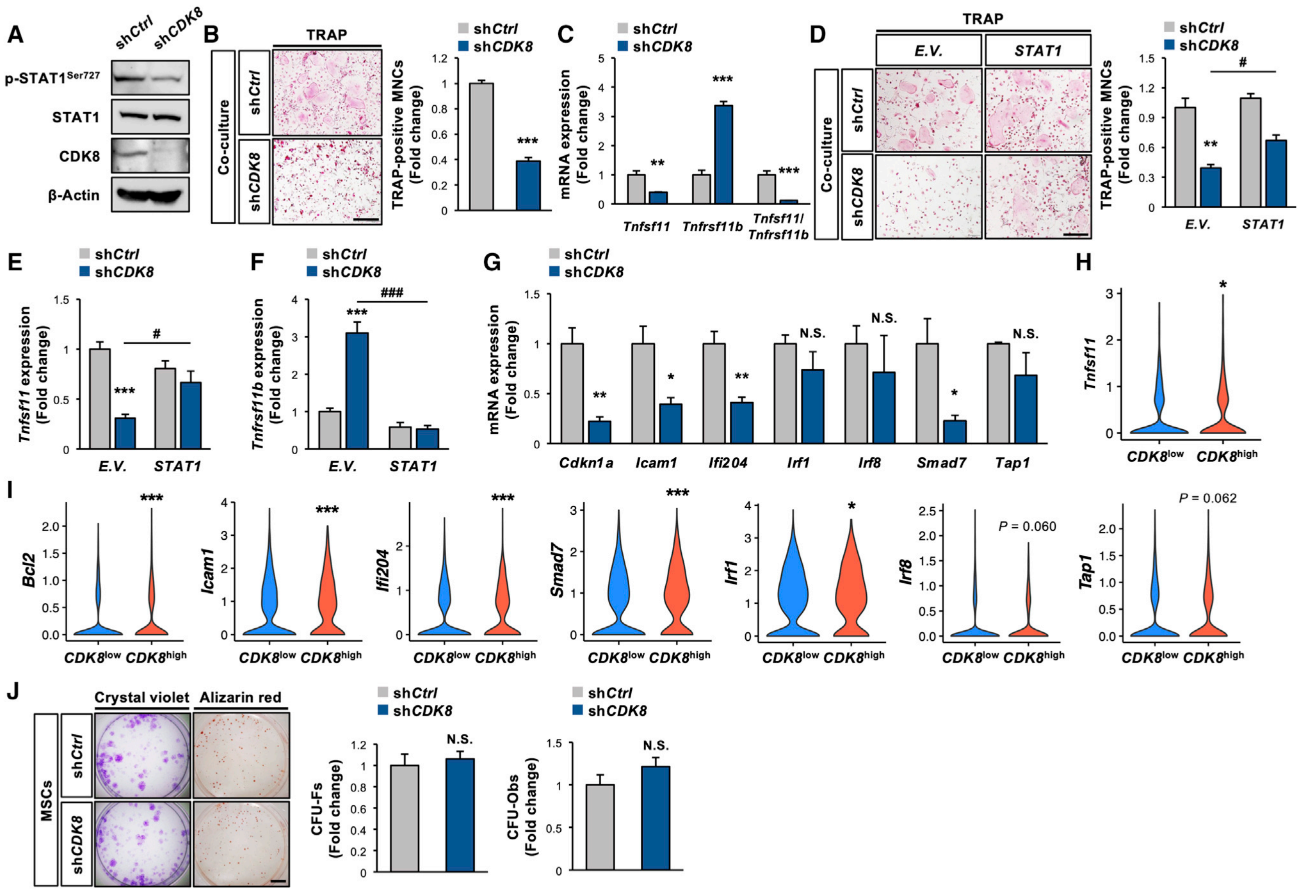

Figure 3. ***CDK8*** **deficiency in MSCs attenuates osteoclastogenesis through STAT1-RANKL axis**
(A) Protein levels of CDK8, p-STAT1^{Ser727}, and STAT1 in MSCs infected with sh*CDK8*. β-Actin served as a loading control.
(B) BMMs prepared from WT mice were co-cultured with MSCs infected with sh*CDK8*, followed by TRAP staining (n = 4 independent replicates, ***p < 0.001).
(C) mRNA levels of *Tnfsf11* and *Tnfrsf11b* and *Tnfsf11*/*Tnfrsf11b* ratio in MSCs infected with sh*CDK8* (n = 4 independent replicates, **p < 0.01, ***p < 0.001).
(D) BMMs prepared from WT mice were co-cultured with MSCs infected with sh*CDK8* in combination with *STAT1* expression vector, followed by TRAP staining (n = 5 independent replicates, **p < 0.01 [sh*Ctrl* + *E.V.* versus sh*CDK8* + *E.V.*], #p < 0.05 [sh*CDK8* + *E.V.* versus sh*CDK8* + *STAT1*]).
(E and F) MSCs were infected with sh*CDK8* in combination with *STAT1* expression vector, followed by determination of mRNA levels of *Tnfsf11* (E) and *Tnfrsf11b* (F) (n = 4 independent replicates, ***p < 0.001 [sh*Ctrl* + *E.V.* versus sh*CDK8* + *E.V.*], #p < 0.05, ###p < 0.001 [sh*CDK8* + *E.V.* versus sh*CDK8* + *STAT1*]).
(G) mRNA levels of STAT1-targeted genes in MSCs were infected with sh*CDK8* (n = 4 independent replicates, *p < 0.05, **p < 0.01).
(H) *Tnfsf11* expression in *CDK8*^{low} (n = 1,185) and *CDK8*^{high} (n = 845) MSCs (*p < 0.05).
(I) mRNA levels of STAT1-targeted genes of *CDK8*^{low} (n = 1,788) and *CDK8*^{high} (n = 1,164) MSCs (*p < 0.05, ***p < 0.001).
(J) MSCs were infected with sh*CDK8*, followed by determination of CFU-Fs and CFU-Obs (n = 3 independent replicates). Scale bars, 800 μm (B and D) and 2 mm (J).
BMM, bone marrow macrophage; CFU-F, colony-forming unit fibroblast; CFU-Ob, colony-forming unit osteoblast; sh*Ctrl*, sh*Control*; E.V., empty vector.

establish the MSC-autonomous nature of the bone resorption phenotype due to the manipulation of *CDK8* expression in MSCs. The CDK8 protein level was markedly reduced by targeting *CDK8* expression using lentiviral shRNA (sh*CDK8*) in MSCs, along with reduced STAT1^{Ser727} phosphorylation levels (Figure 3A). Co-culture of sh*CDK8*-infected MSCs and wild-type (WT) mice-derived bone marrow-derived macrophages (WT-BMMs) generated fewer multinucleated osteoclasts than the co-culture of sh*Control*-infected MSCs and WT-BMMs

(Figure 3B). We confirmed that the repression of osteoclastogenesis was also observed by co-culture of MSCs isolated from *Prx1-Cre;CDK8*$^{fl/fl}$ mice and WT-BMMs (Figure S2A). The level of *Tnfsf11*, encoding pro-osteoclastogenic cytokine RANKL, was markedly decreased in *CDK8*-deficient MSCs, whereas *Tnfrsf11b*, encoding the anti-osteoclastogenic cytokine osteoprotegerin, was significantly increased, concomitant with a significant decrease in the *Tnfsf11*/*Tnfrsf11b* ratio (Figure 3C). Previous reports demonstrated that STAT1, a direct target of CDK8, transcriptionally controls *Tnfsf11* expression (Sundaram et al., 2009); thus, whether STAT1 may contribute to the mechanisms underlying the extrinsic regulation of osteoclastogenesis by CDK8 in MSCs remains to be elucidated. *STAT1* overexpression significantly attenuated the osteoclastogenesis suppression by *CDK8* deficiency in MSCs, with the correction of changes in *Tnfsf11* and *Tnfrsf11b* levels by *CDK8* deficiency (Figures 3D–3F and S2B). We confirmed the augmentation of STAT1-dependent signal (STAT1^{Ser727} phosphorylation and STAT1-dependent transcription) by STAT1 overexpression in MSCs irrespective of CDK19 deficiency (Figures S2C and S2D). The expression levels of STAT1-targeted genes such as *Cdkn1a*, *Icam1*, *Ifi204*, and *Smad7* were significantly decreased by *CDK8* deficiency in MSCs (Figure 3G), but the expression level of *Spi1* (encoding PU.1), which is controlled by CDK8 (Amirhosseini et al., 2019), was not significantly altered by *CDK8* deficiency (Figure S2E). In line with our *in vitro* studies, an analysis of the scRNA-seq dataset revealed that the levels of *Tnfsf11* and STAT1-targeted genes such as *Bcl2*, *Icam1*, *Ifi204*, *Smad7*, and *Irf1* were significantly higher in *CDK8*^{high} MSCs than in *CDK8*^{low} MSCs (Figures 3H and 3I). Conversely, *CDK8* deficiency did not significantly affect colony-forming unit fibroblasts (CFU-Fs) and CFU osteoblasts (CFU-Obs) in MSCs (Figure 3J).

Collectively, these results indicate that the CDK8-STAT1 axis in MSCs could be a critical pathway for the extrinsic regulation of osteoclastogenesis rather than the intrinsic regulation of their stemness or osteogenic potential.

CDK8 deficiency in MSCs corrects age-associated enhancement of their osteoclastogenesis-supporting activity

The fact that aging increases the *CDK8* level in MSCs in association with aging-related signals (Figure 1) and *CDK8*-deficient MSCs are less supportive of osteoclastogenesis (Figures 2 and 3) led us to investigate whether aged MSCs could have high osteoclastogenesis-supporting activity by a CDK8-dependent manner. To understand the properties of aged MSCs, their senescence, stemness, and osteogenic potential *in vitro* should be examined. As anticipated, the number of senescence-associated β-galactosidase-positive cells was significantly higher in aged MSCs obtained from 16-month-old mice than young MSCs from 3-month-old mice (Figure 4A). Moreover, both CFU-Fs and CFU-Obs were more significantly reduced in aged MSCs than that in young MSCs (Figure 4B). Together, aged MSCs showed increased senescence and decreased stemness and osteogenic potential under our experimental conditions. WT-BMM co-culture with aged MSCs generated significantly greater multinucleated osteoclasts than that with young MSCs, along with significant increases in the level of *Tnfsf11* and the *Tnfsf11*/*Tnfrsf11b* ratio, indicating that aged MSCs have a higher ability to support osteoclastogenesis rather than that of young MSCs (Figures 4C and 4D). Furthermore, *CDK8* silencing in aged MSCs significantly repressed the age-associated increase in the generation of multinucleated osteoclasts, along with marked decreases in STAT1^{Ser727} phosphorylation and CDK8 levels (Figures 4E and 4F).

These results indicate that CDK8 in MSCs could be implicated in their high osteoclastogenesis-supporting activity associated with aging.

Pharmacological inhibition of CDK8 represses MSC-dependent osteoclastogenesis and prevents ovariectomy-induced osteoclastic activation and bone loss

We recently identified 4-acetyl-3-{4-[2-(tetrahydropyran-4-yloxy)ethoxy]phenoxy}benzamide (hereafter referred to as KY-065) as a novel potential CDK8 inhibitor, which inhibits the stemness and tumorigenicity of glioma stem cells, thus exerting anti-glioblastoma potential *in vivo* (Fukasawa et al., 2021). KY-065 did not significantly affect the osteoclastogenesis of BMMs and the expression of osteoclastic marker genes (*Spi1*, *Ctsk*, *Acp5*, and *Mmp9*), irrespective of a marked decrease in the STAT1^{Ser727} phosphorylation level (Figures 5A, 5B, and S3A), indicating that pharmacological inhibition of CDK8 by KY-065 had no inhibitory effect on intrinsic osteoclastogenesis. As observed by the genetic inhibition of *CDK8* in MSCs (Figure 3), neither the stemness nor the osteogenic potential of MSCs was changed by the KY-065 treatment, irrespective of the marked decrease in the phosphorylation level of STAT1^{Ser727} (Figures 5C–5E). Given that genetic inhibition of *CDK8* in MSCs increased the bone mass due to suppressed osteoclastic activity *in vivo* (Figure 2), whether the pharmacological inhibition of CDK8 in MSCs by KY-065 could prevent osteoclastic activation and bone loss was examined next. KY-065 treatment significantly decreased extrinsic osteoclastogenesis derived from the co-culture system using WT mice-derived MSCs (WT-MSCs) and WT-BMMs, along with decreases in the *Tnfsf11* level and the *Tnfsf11/Tnfrsf11b* ratio in WT-MSCs (Figures 5F and 5G), indicating that KY-065 could inhibit

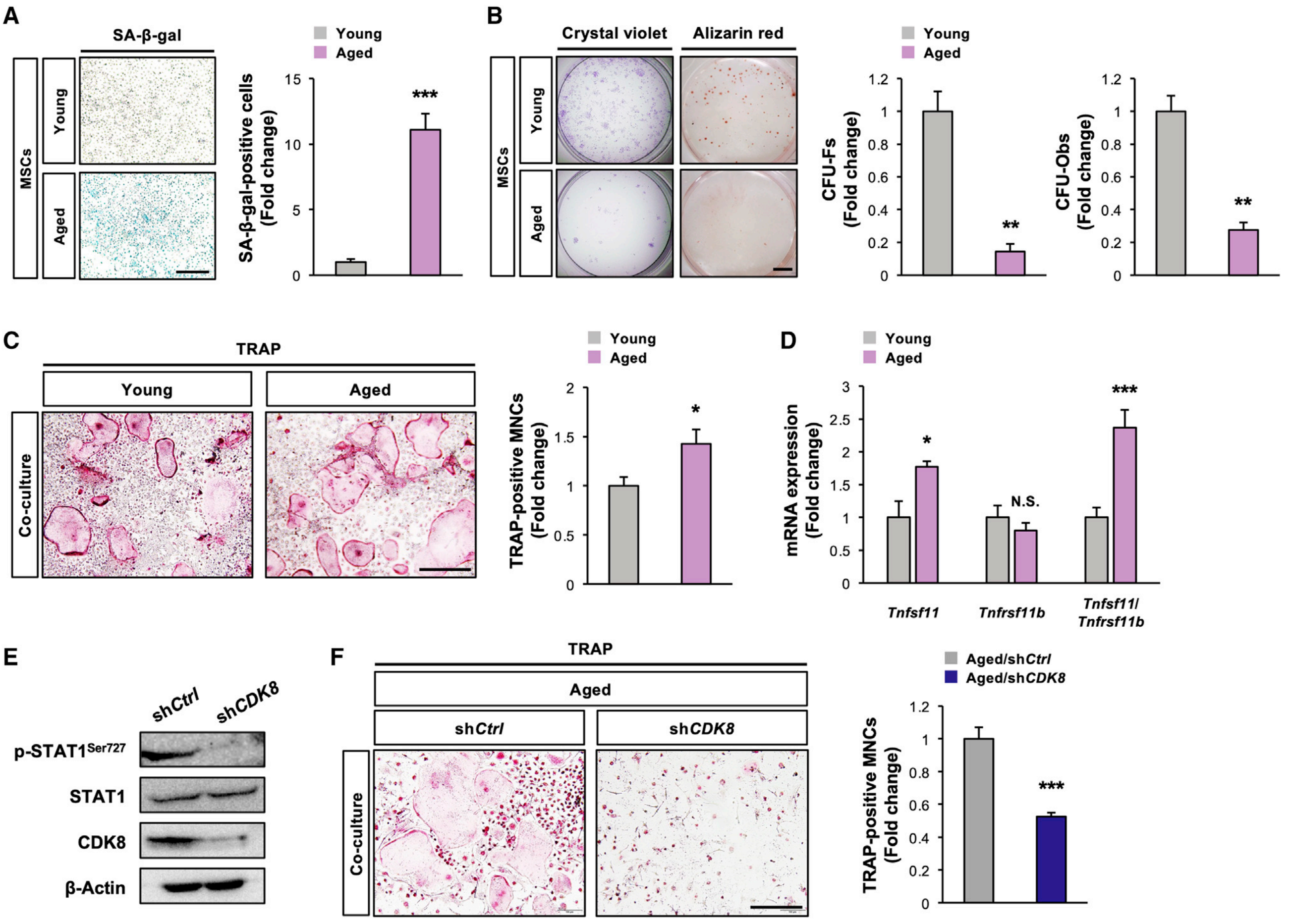

Figure 4. *CDK8* deficiency in MSCs corrects higher osteoclastogenesis-supporting activity associated with aging

(A and B) Aged MSCs were cultured, followed by determination of (A) SA-β-gal activity (n = 4 independent replicates, $***p < 0.001$) and (B) CFU-Fs and CFU-Obs (n = 3 independent replicates, $**p < 0.01$).

(C) BMMs prepared from WT mice were co-cultured with aged MSCs, followed by TRAP staining (n = 4 independent replicates, $*p < 0.05$).

(D) mRNA levels of *Tnfsf11* and *Tnfrsf11b* and *Tnfsf11*/*Tnfrsf11b* ratio in aged MSCs (n = 4 independent replicates, $*p < 0.05$, $***p < 0.001$).

(E) Protein levels of CDK8, p-STAT1^{Ser727}, and STAT1 in aged MSCs infected with sh*CDK8*. β-Actin served as a loading control.

(F) BMMs prepared from WT mice were co-cultured with aged MSCs infected with sh*CDK8*, followed by TRAP staining (n = 4 independent replicates, $***p < 0.001$).

Scale bars, 800 μm (A, C, and F) and 2 mm (B). MSC, mesenchymal stem cell; SA-β-gal, senescence-associated β-galactosidase; CFU-F, colony-forming unit fibroblast; CFU-Ob, colony-forming unit osteoblast; BMM, bone marrow macrophage; sh*Ctrl*, sh*Control*.

the osteoclastogenesis-supporting activity of MSCs. Next, whether KY-065 could prevent the ovariectomy-induced osteoclastic activation and bone loss *in vivo* was determined. KY-065 was subcutaneously administered to ovariectomized mice for 28 consecutive days. KY-065 did not significantly affect the ovariectomy-induced loss of uterine weight (data not shown). KY-065 administration significantly suppressed the reduction in bone volume in the cancellous bone of ovariectomized mice (Figure 5H). Furthermore, histomorphometric analysis revealed that KY-065 administration significantly inhibited the ovariectomy-induced increases in the bone resorption parameters (osteoclast surface and number) without affecting the bone formation parameters (Figure 5I). In accordance with pharmacological inhibition by KY-065, ovariectomy-induced bone loss and osteoclast activation were significantly repressed in *Prx1-Cre;CDK8$^{fl/fl}$* mice (Figures S3B and S3C).

Collectively, these results demonstrate that KY-065, a small-molecule CDK8 inhibitor, could inhibit osteoclastic activation and bone loss possibly in a non-cell-autonomous manner (acting on MSCs) rather than a cell-autonomous manner (acting on BMMs or osteoclasts), thus producing an anti-osteoporotic potential *in vivo*.

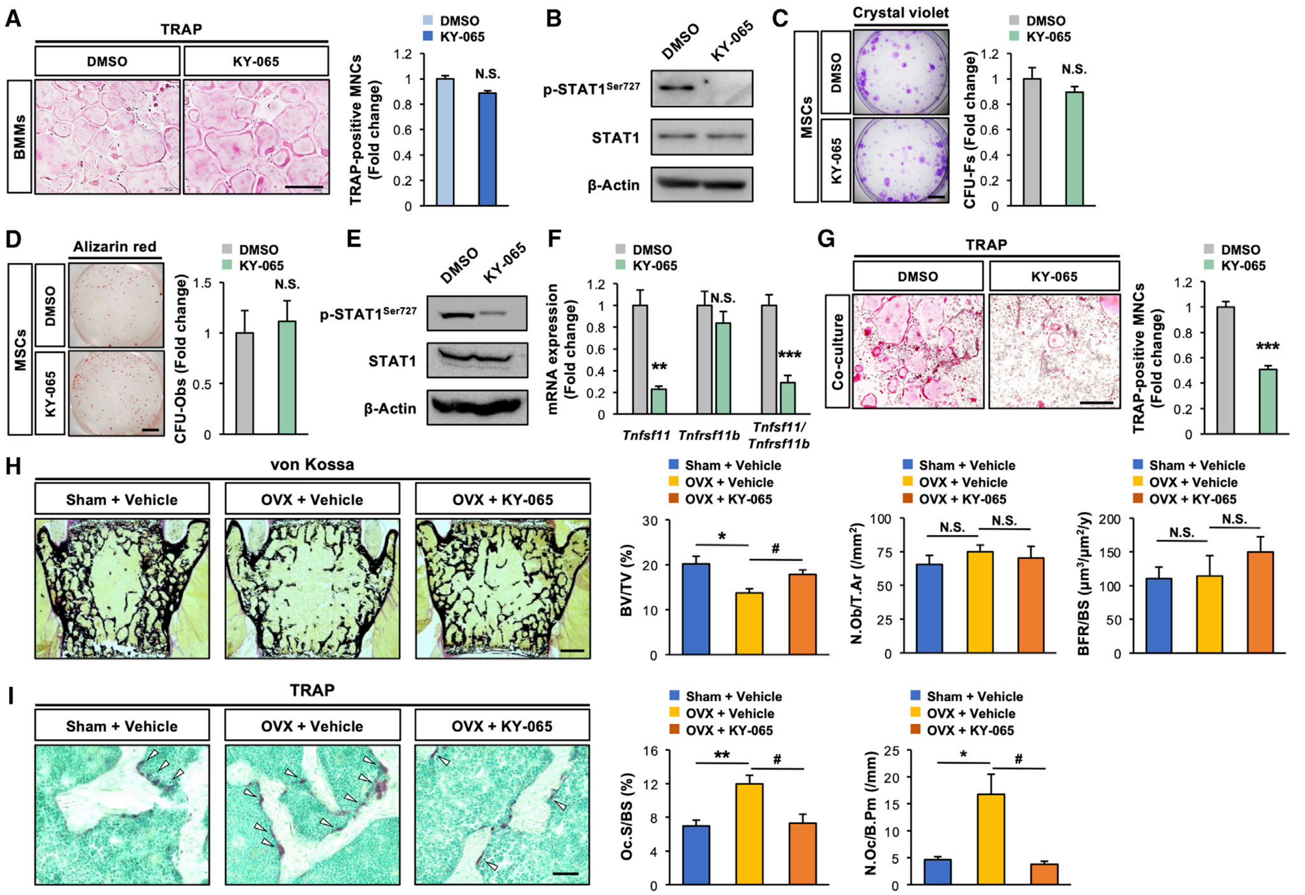

Figure 5. Pharmacological inhibition of CDK8 in MSCs decreases their osteoclastogenesis-supporting activity and protects against OVX-mediated bone loss

(A and B) BMMs prepared from WT mice were stimulated with RANKL in the presence of 30 nM KY-065, followed by (A) TRAP staining (n = 3 independent replicates) and (B) determination of protein levels of p-STAT1^{Ser727} and STAT1. β-Actin served as a loading control.

(C–E) MSCs were treated with 30 nM KY-065, followed by determination of (C) CFU-Fs (n = 3 independent replicates), (D) CFU-Obs (n = 3 independent replicates), and (E) protein levels of p-STAT1^{Ser727} and STAT1. β-Actin served as a loading control.

(F) mRNA levels of *Tnfsf11* and *Tnfrsf11b* and *Tnfsf11/Tnfrsf11b* ratio in MSCs treated with 30 nM KY-065 (n = 4 independent replicates, $^{**}p < 0.01$, $^{***}p < 0.001$).

(G) BMMs prepared from WT mice were co-cultured with MSCs in the presence of 30 nM KY-065, followed by TRAP staining (n = 4 independent replicates, $^{***}p < 0.001$).

(H) BV/TV measured by von Kossa staining, N.Ob/T. Ar determined by toluidine blue staining and BFR/BS determined by calcein labeling of vertebrae (n = 10, $^{*}p < 0.05$ [sham + vehicle versus OVX + vehicle], $^{\#}p < 0.05$ [OVX + vehicle vs OVX + KY-065]).

(I) Oc.S/BS and N.Oc/B.Pm determined by TRAP staining of femur (n = 10, $^{**}p < 0.01$ [sham + vehicle versus OVX + vehicle], $^{\#}p < 0.05$ [OVX + vehicle versus OVX + KY-065]). Arrowheads indicate TRAP-positive osteoclasts.

Scale bars, 800 μm (A and G), 2 mm (C and D), 500 μm (H), and 50 μm (I). OVX, ovariectomy.

DISCUSSION

MSCs are known to function as a reservoir of osteoblasts that regulate postnatal bone homeostasis throughout the lifespan, with functions that gradually decreased with aging, leading to osteoporosis and susceptible to fracture over time (Rachner et al., 2011; Zhou et al., 2014). Aging has been reported to significantly increase MSC/osteoblastic cell-dependent osteoclastogenesis (Cao et al., 2005) and exosomal miR-31a-5p derived from aged MSCs increasing osteoclastogenesis in aging bone tissue (Xu et al., 2018), although molecular mechanisms underlying the communication between osteoclasts and MSCs are not yet fully understood. The main relevance of our findings is that CDK8, a transcription-related CDK, is highly expressed in aged MSCs and is functionally essential for bone remodeling by controlling bone resorption, at least in part in trabecular bone in young mice, through its expression in

MSCs. Further examination is required to investigate the role of CDK8 in cortical bone as well as in trabecular bone of aged mice, because previous studies showed that osteoclasts increase in the endocortical surfaces of long bones but not in trabecular bone in aged mice, and RANKL from osteoblasts/osteocytes is indispensable for the increase in osteoclasts and loss of cortical bone with age (Almeida et al., 2007; Farr et al., 2017; Kim et al., 2020; Piemontese et al., 2017). Although omics analyses may reveal the additional molecular bases by which CDK8 in MSCs influence bone homeostasis, to the best of our knowledge, this is the first study to show that CDK8 in MSCs acts non-autonomously to regulate osteoclastogenesis and bone resorption, without affecting the cell-autonomous function of MSCs such as their stemness and osteogenic potential.

CDK8 deficiency in MSCs (*Prx1-Cre;CDK8$^{fl/fl}$* mice) leads to high bone mass with lower bone resorption indices, while *CDK8* deficiency in osteoprogenitors (*Osx-Cre;CDK8$^{fl/fl}$* mice) and osteoblasts (*Col1a1-Cre;CDK8$^{fl/fl}$* mice) showed normal bone mass with normal osteoclasts. *CDK8* should be ablated in MSCs and their descendants of *Prx1-Cre;CDK8$^{fl/fl}$* mice. On the contrary, *CDK8* should be deleted in osteoprogenitors and their descendants of *Osx-Cre;CDK8$^{fl/fl}$* mice, and in osteoblasts and their descendants of *Col1a1-Cre;CDK8$^{fl/fl}$* mice. Accordingly, *CDK8* deficiency in MSCs should be observed only in *Prx1-Cre;CDK8$^{fl/fl}$* mice, among the mutant mice generated in this study, suggesting that CDK8 may have a pivotal role in bone resorption and homeostasis through its expression in MSCs rather than osteoprogenitors or osteoblasts *in vivo*, although immunostaining comparing the loss of CDK8 protein in these cell types of these mutant mice should be performed.

Osteoporosis is an enormous public health problem that will only increase in scope with the aging population (Curtis and Safford, 2012). Regarding the treatment of osteoporosis, bisphosphonates and recombinant human parathyroid hormone produce their therapeutic effects via either anabolic or anti-resorptive function, with limitation of long-term treatment due to their adverse effects (Kennel and Drake, 2009; Ponnapakkam et al., 2014; Reid, 2015). We found that the pharmacological inhibition of CDK8, the novel small-molecule CDK8 inhibitor KY-065, effectively attenuates the MSC-autonomous nature of the bone resorption phenotype, which was consistent with the data obtained from the genetic inhibition of *CDK8* *in vitro* and *in vivo*. Because ovariectomy decreases the apoptosis of osteoclasts, which contributes to the enhancement of bone resorption (Feng and Teitelbaum, 2013), the present data that KY-065 prevents ovariectomy-induced increases in both osteoclast surface and osteoclast number may indicate that the pharmacological inhibition of CDK8 could accelerate osteoclastic apoptosis as well as inhibit osteoclastic differentiation. Previous studies have shown that the conditional inducible deletion of *CDK8* in adult mice exhibits no gross or histopathological defects in many tissues (McCleland et al., 2015), although a global *CDK8* knockout leads to embryonic lethality due to a pre-implantation defect (Westerling et al., 2007), suggesting that KY-065 treatment will not necessarily cause severe side effects during osteoporosis therapy in adults. Collectively, our findings suggest that CDK8 may be considered an attractive treatable target against various metabolic bone diseases relevant to abnormal osteoclastogenesis, including age-related osteoporosis and postmenopausal osteoporosis.

EXPERIMENTAL PROCEDURES

Mice

CDK8-floxed line was obtained from the EUCOMM Consortium. *CDK8$^{fl/fl}$* mice were crossed with either *Prx1-Cre*, *Col1a1-Cre*, or *Osx-Cre* mice (Dacquin et al., 2002; Logan et al., 2002; Menzl et al., 2019; Rodda and McMahon, 2006). These mutant mice were backcrossed more than five generations with C57BL/6J mice. Genotyping was performed by PCR using tail genomic DNA with specific primers (Table S1). Genomic deletions were detected by PCR with specific primers (Table S2). Mice were bred under standard animal housing conditions at 23°C ± 1°C with a relative humidity of 55% and a light/dark cycle of 12 h, with free access to food and water. The study protocol meets the guidelines of the Japanese Pharmacological Society and was approved by the Committee for the Ethical Use of Experimental Animals at Gifu Pharmaceutical University and Gifu University. The number of animals used per experiment is stated in the figure legends.

Ovariectomy and KY-065 treatment

Female ddY mice were purchased from Japan SLC. These mice were subjected to ovariectomy or sham operation under aseptic environments using three types of mixed anesthetic agents at 8 weeks old, as previously described (Hinoi et al., 2007). Ovariectomized ddY mice were also subjected to subcutaneous injection of KY-065, dissolved in saline with 8.4% hydroxypropyl β-cyclodextrin, twice per day for 28 consecutive days at a dose of 30 mg/kg. Hydroxypropyl β-cyclodextrin is a solubilizer for KY-065 and was used as a vehicle. The mice were killed by decapitation at 28 days after the operation, followed by dissection of their vertebra and femur.

Bone histomorphometric and μCT analysis

Bone histomorphometric analyses were performed on femurs and vertebrae (Yamamoto et al., 2012). Briefly, the vertebrae were fixed with 70% ethanol, followed by dehydration in an ethanol series and subsequent embedding in methyl methacrylate resin. The femurs were fixed with 4% paraformaldehyde, followed by infiltration in sucrose gradient and subsequent embedding in O.C.T. Compound (Tissue-Tek). BV/TV was measured by von Kossa

staining. Osteoblast and osteoclast parameters were analyzed by toluidine blue staining and TRAP staining, respectively. Calcein was intraperitoneally injected twice with an interval of 3 days, and then, mice were killed 2 days after the last injection. Quantification was performed using the OsteoMeasure analysis system (OsteoMetrics). Trabecular architecture of femur was assessed using a μCT system (Comscan Techno) at 90 kV and 45 μA, and BV/TV was measured using TRI/3D-BON software (RATOC) (Ozaki et al., 2019).

Measurement of serum TRAP activity

The serum was mixed with TRAP assay buffer (17.6 mg/mL L-ascorbic acid, 9.2 mg/mL sodium tartrate dihydrate, 3.6 mg/mL *p*-nitrophenyl phosphoric acid disodium salt, 0.3% Triton X-100, 6 mM EDTA, 600 mM NaCl, 600 mM sodium acetate, pH 5.5). Samples were then incubated at 37°C for 30 min, followed by measurement of the absorbance at 405 nm using the GloMax-Multi Detection System (Promega).

Cell culture

HEK293T cells were purchased from RIKEN Cell Bank. These cells were cultured at 37°C in 5% CO_2 and maintained in Dulbecco's modified Eagle's medium (DMEM) supplemented with 10% fetal bovine serum (FBS) and 1% penicillin/streptomycin.

Primary bone marrow MSCs were isolated as follows. Briefly, the femur and tibia were isolated and soft tissue was removed. The epiphyses were removed and bone marrow cells were collected by centrifugation. Red blood cells were lysed with ammonium chloride. Cells were cultured at a density of 4×10^6 cells/well in 6-well plates with α-MEM supplemented with 20% FBS and 1% penicillin/streptomycin at 37°C in 5% CO_2. For CFU-F assays, after 14 days in culture, CFU-F colonies were stained by crystal violet solution and counted. For CFU-Ob assays, after 5 days in culture, osteoblast differentiation was initiated by replacing the medium with α-MEM supplemented with 10% FBS, 50 μg/mL ascorbic acid, 10 mM β-glycerophosphate, and 1% penicillin/streptomycin for 14 days. CFU-Ob colonies were assessed by alizarin red staining. Aged MSCs and young MSCs were isolated from 18-month-old mice and 4-week-old mice, respectively.

Bone marrow cells obtained from the long bones were stimulated with 100 ng/mL macrophage colony-stimulating factor (M-CSF) for 3 days in α-MEM supplemented with 10% FBS and 1% penicillin/streptomycin; we used these cells as bone marrow macrophages (BMMs). BMMs were cultured in α-MEM supplemented with 10% FBS, 20 ng/mL M-CSF, 20 ng/mL RANKL, and 1% penicillin/streptomycin for 5 days, followed by TRAP staining, as previously described (Fukasawa et al., 2016). For co-culture, MSCs were cultured with BMMs in α-MEM supplemented with 10% FBS, 10 nM 1,25-dihydroxycholecalciferol (vitamin D_3), and 1% penicillin/streptomycin for 8 days, followed by TRAP staining. Senescence-associated β-galactosidase (SA-β-gal) activity was detected with a Senescent Cells Histochemical Staining Kit (#CS0030, Sigma-Aldrich) according to the manufacturer's instructions. Cell size was determined by measuring mean forward scatter area (FSC-A) using flow cytometer CytoFLEX S (Beckman Coulter).

Lentiviral transfection

Vectors were transfected into HEK293T cells using the calcium phosphate method. Virus supernatants were collected 48 h after transfection and then cells were infected with virus supernatants for 24 h in the presence of 2 μg/mL polybrene (Iezaki et al., 2018a). Cells were then subjected to selection by culture with 1 μg/mL puromycin for 3 days before usage for experiments. Plasmids pLKO.1.sh*CDK8* #1 (#19760, deposited by William Hahn), pLKO.1 puro (#8453, deposited by Bob Weinberg), and FLAG-STAT1 (#71454, deposited by George Stark) were obtained from Addgene. pLKO.1.sh*CDK19* (#TRCN0000003140) was purchased from Sigma-Aldrich.

Immunoblotting analysis

Cultured cells were solubilized in lysis buffer (10 mM Tris-HCl, 150 mM NaCl, 0.5 mM EDTA, 10 mM NaF, 1% Nonidet P-40, pH 7.4) containing protease inhibitor cocktail. Samples were then subjected to SDS-PAGE, followed by transfer to polyvinylidene fluoride (PVDF) membranes and subsequent immunoblotting assay (Iezaki et al., 2018b). The primary antibodies used were anti-CDK8 (1:1,000, #4101), anti-Stat1 (1:1,000, #9172), anti-phospho-Stat1 (S727) (1:1,000, #9177), anti-Stat3 (1:1,000, #9132), and anti-phospho-Stat3 (S727) (1:1,000, #9134) (all from Cell Signaling Technologies), and anti-β-actin (1:2,000, #4778, Santa Cruz Biotechnology). Primary antibodies were diluted with blocking solution (5% skim milk). Quantification was performed by densitometry using ImageJ (NIH).

Quantitative real-time-PCR

Total RNA was extracted from cells, followed by synthesis of cDNA with reverse transcriptase and oligo-dT primer. The cDNA samples were then used as templates for real-time PCR analyses, which were performed on an MX3005P instrument (Agilent Technologies), by using specific primers for each gene (Table S3). Expression levels of the genes examined were normalized by using the *Gapdh* expression levels as an internal control for each sample (Iezaki et al., 2016).

Luciferase assay

Cells were transfected with gamma-activated sequence (GAS) reporter vector using the lipofection method, as previously described (Horie et al., 2022), followed by the preparation of cell lysates and subsequent determination of luciferase activity using specific substrates in a GloMax-Multi Detection System (Promega).

scRNA-seq data analysis

We obtained expression data of scRNA-seq (Database: GSE145477) (Zhong et al., 2020) from GEO (https://www.ncbi.nlm.nih.gov/geo/) and used the Seurat R package. Cells expressing more than 6,000 or less than 200 genes and more than 5% of mitochondrial genes were defined as poor-quality data and these data were excluded. After normalization and scaling, we performed dimensionality reduction by principal-component analysis (PCA) and visualized using t-SNE. We used the Leiden algorithm for clustering and assigned cell labels based on well-known cell-specific marker genes. During clustering, blood cells, endothelial cells, pericytes, and adipocytes were removed. Differential expressed genes were

determined by the Wilcoxon rank-sum test. For GSEA, we imported scRNA-seq data (Seurat object) into the Presto R package and calculated area under the receiver operator curve (AUC) to generate the gene list. GSEA was performed using the clusterProfiler R package.

Bulk RNA-seq data analysis

Raw RNA-seq data (Database: GSE133922) (Helbling et al., 2019) were downloaded from GEO using the SRA Toolkit (version 2.10.4). Quality check of fastq files and trimming of adaptors/low-quality reads were performed using FastQC (version 0.11.8) and Cutadapt (version 2.10), respectively. Then, we aligned sequence reads with STAR (version 2.6.1c) against the mouse reference sequence (GRCm38) and calculated transcripts per million (TPM) using RSEM (version 1.3.1). Gene expression levels were represented as log2-transformed TPM values. Statistical analysis was performed using the Wilcoxon rank-sum test.

Statistical analysis

Unless otherwise specified, the results are expressed as the means ± standard error (SE), and statistical significance was determined by the two-tailed Student's *t* test or Bonferroni test for multiple comparisons. $p < 0.05$ was considered statistically significant.

SUPPLEMENTAL INFORMATION

Supplemental information can be found online at https://doi.org/10.1016/j.stemcr.2022.06.001.

AUTHOR CONTRIBUTIONS

E.H. and T.Y. conceived and designed the study. T.Y., T. Kadota, J.L., S.O., S.I., G.P., R.U., and T.I. performed the *in vivo* experiments. K.F., K.T., and A.S. performed the *in vitro* experiments. T.H. performed the bioinformatic analysis. M.Y., T. Kitao, H.O., S.S., and H.S. discussed the results and provided critical reagents and comments. E.H. and T.Y. wrote the manuscript. All of the authors commented on the manuscript and approved the manuscript.

ACKNOWLEDGMENTS

This work was supported in part by the Japan Society for the Promotion of Science (16H05131, 17KT0051 and 18H04971, to E.H.), and a grant from the Lotte Foundation (to E.H.). Bioinformatic analyses were performed using the super-computing resource provided by Human Genome Center, Institute of Medical Science, University of Tokyo.

CONFLICTS OF INTERESTS

T. Kadota, M.Y., T. Kitao, and H.S. are employees of Kyoto Pharmaceutical Industries. E.H. is supported by a research fund from Kyoto Pharmaceutical Industries. The other authors declare no competing interests.

Received: June 22, 2021
Revised: June 1, 2022
Accepted: June 2, 2022
Published: June 30, 2022

REFERENCES

Almeida, M., Han, L., Martin-Millan, M., Plotkin, L.I., Stewart, S.A., Roberson, P.K., Kousteni, S., O'Brien, C.A., Bellido, T., Parfitt, A.M., et al. (2007). Skeletal involution by age-associated oxidative stress and its acceleration by loss of sex steroids. J. Biol. Chem. *282*, 27285–27297. https://doi.org/10.1074/jbc.M702810200.

Ambrosi, T.H., Scialdone, A., Graja, A., Gohlke, S., Jank, A.M., Bocian, C., Woelk, L., Fan, H., Logan, D.W., Schürmann, A., et al. (2017). Adipocyte accumulation in the bone marrow during obesity and aging impairs stem cell-based hematopoietic and bone regeneration. Cell Stem Cell *20*, 771–784.e6. https://doi.org/10.1016/j.stem.2017.02.009.

Amirhosseini, M., Bernhardsson, M., Lång, P., Andersson, G., Flygare, J., and Fahlgren, A. (2019). Cyclin-dependent kinase 8/19 inhibition suppresses osteoclastogenesis by downregulating RANK and promotes osteoblast mineralization and cancellous bone healing. J. Cell. Physiol. *234*, 16503–16516. https://doi.org/10.1002/jcp.28321.

Bancerek, J., Poss, Z.C., Steinparzer, I., Sedlyarov, V., Pfaffenwimmer, T., Mikulic, I., Dölken, L., Strobl, B., Müller, M., Taatjes, D.J., and Kovarik, P. (2013). CDK8 kinase phosphorylates transcription factor STAT1 to selectively regulate the interferon response. Immunity *38*, 250–262. https://doi.org/10.1016/j.immuni.2012.10.017.

Cao, J.J., Wronski, T.J., Iwaniec, U., Phleger, L., Kurimoto, P., Boudignon, B., and Halloran, B.P. (2005). Aging increases stromal/osteoblastic cell-induced osteoclastogenesis and alters the osteoclast precursor pool in the mouse. J. Bone Miner. Res. *20*, 1659–1668. https://doi.org/10.1359/jbmr.050503.

Cauley, J.A. (2013). Public health impact of osteoporosis. J Gerontol. A Biol. Sci. Med. Sci. *68*, 1243–1251. https://doi.org/10.1093/gerona/glt093.

Curtis, J.R., and Safford, M.M. (2012). Management of osteoporosis among the elderly with other chronic medical conditions. Drugs Aging *29*, 549–564. https://doi.org/10.2165/11599620-000000000-00000.

Dacquin, R., Starbuck, M., Schinke, T., and Karsenty, G. (2002). Mouse alpha1(I)-collagen promoter is the best known promoter to drive efficient Cre recombinase expression in osteoblast. Dev. Dyn. *224*, 245–251. https://doi.org/10.1002/dvdy.10100.

Carbonare, L.D., Valenti, M.T., Zanatta, M., Donatelli, L., and Lo Cascio, V. (2009). Circulating mesenchymal stem cells with abnormal osteogenic differentiation in patients with osteoporosis. Arthritis Rheum. *60*, 3356–3365. https://doi.org/10.1002/art.24884.

Farr, J.N., Xu, M., Weivoda, M.M., Monroe, D.G., Fraser, D.G., Onken, J.L., Negley, B.A., Sfeir, J.G., Ogrodnik, M.B., Hachfeld, C.M., et al. (2017). Targeting cellular senescence prevents age-related bone loss in mice. Nat. Med. *23*, 1072–1079. https://doi.org/10.1038/nm.4385.

Feng, X., and McDonald, J.M. (2011). Disorders of bone remodeling. Annu. Rev. Pathol. *6*, 121–145. https://doi.org/10.1146/annurev-pathol-011110-130203.

Feng, X., and Teitelbaum, S.L. (2013). Osteoclasts: new insights. Bone Res. *1*, 11–26. https://doi.org/10.4248/br201301003.

Fukasawa, K., Kadota, T., Horie, T., Tokumura, K., Terada, R., Kitaguchi, Y., Park, G., Ochiai, S., Iwahashi, S., Okayama, Y., et al. (2021). CDK8 maintains stemness and tumorigenicity of glioma stem cells by regulating the c-MYC pathway. Oncogene *40*, 2803–2815. https://doi.org/10.1038/s41388-021-01745-1.

Fukasawa, K., Park, G., Iezaki, T., Horie, T., Kanayama, T., Ozaki, K., Onishi, Y., Takahata, Y., Yoneda, Y., Takarada, T., et al. (2016). ATF3 controls proliferation of osteoclast precursor and bone remodeling. Sci. Rep. *6*, 30918. https://doi.org/10.1038/srep30918.

Harada, S.-i., and Rodan, G.A. (2003). Control of osteoblast function and regulation of bone mass. Nature *423*, 349–355. https://doi.org/10.1038/nature01660.

Helbling, P.M., Piñeiro-Yáñez, E., Gerosa, R., Boettcher, S., Al-Shahrour, F., Manz, M.G., and Nombela-Arrieta, C. (2019). Global transcriptomic profiling of the bone marrow stromal microenvironment during postnatal development, aging, and inflammation. Cell Rep. *29*, 3313–3330.e4. https://doi.org/10.1016/j.celrep.2019.11.004.

Hinoi, E., Takarada, T., Uno, K., Inoue, M., Murafuji, Y., and Yoneda, Y. (2007). Glutamate suppresses osteoclastogenesis through the cystine/glutamate antiporter. Am. J. Pathol. *170*, 1277–1290. https://doi.org/10.2353/ajpath.2007.061039.

Horie, T., Fukasawa, K., Yamada, T., Mizuno, S., Iezaki, T., Tokumura, K., Iwahashi, S., Sakai, S., Suzuki, A., Kubo, T., et al. (2022). Erk5 in bone marrow mesenchymal stem cells regulates bone homeostasis by preventing osteogenesis in adulthood. Stem Cell. *40*, 411–422. https://doi.org/10.1093/stmcls/sxac011.

Iezaki, T., Fukasawa, K., Horie, T., Park, G., Robinson, S., Nakaya, M., Fujita, H., Onishi, Y., Ozaki, K., Kanayama, T., et al. (2018a). The MAPK Erk5 is necessary for proper skeletogenesis involving a Smurf-Smad-Sox9 molecular axis. Development *145*, dev164004. https://doi.org/10.1242/dev.164004.

Iezaki, T., Fukasawa, K., Park, G., Horie, T., Kanayama, T., Ozaki, K., Onishi, Y., Takahata, Y., Nakamura, Y., Takarada, T., et al. (2016). Transcriptional modulator Ifrd1 regulates osteoclast differentiation through enhancing the NF-κB/NFATc1 pathway. Mol. Cell Biol. *36*, 2451–2463. https://doi.org/10.1128/mcb.01075-15.

Iezaki, T., Horie, T., Fukasawa, K., Kitabatake, M., Nakamura, Y., Park, G., Onishi, Y., Ozaki, K., Kanayama, T., Hiraiwa, M., et al. (2018b). Translational control of Sox9 RNA by mTORC1 contributes to skeletogenesis. Stem Cell Rep. *11*, 228–241. https://doi.org/10.1016/j.stemcr.2018.05.020.

Karsenty, G., Kronenberg, H.M., and Settembre, C. (2009). Genetic control of bone formation. Annu. Rev. Cell Dev. Biol. *25*, 629–648. https://doi.org/10.1146/annurev.cellbio.042308.113308.

Kennel, K.A., and Drake, M.T. (2009). Adverse effects of bisphosphonates: implications for osteoporosis management. Mayo Clin. Proc. *84*, 632–637. https://doi.org/10.1016/s0025-6196(11)60752-0.

Kfoury, Y., and Scadden, D.T. (2015). Mesenchymal cell contributions to the stem cell niche. Cell Stem Cell *16*, 239–253. https://doi.org/10.1016/j.stem.2015.02.019.

Kim, H.N., Xiong, J., MacLeod, R.S., Iyer, S., Fujiwara, Y., Cawley, K.M., Han, L., He, Y., Thostenson, J.D., Ferreira, E., et al. (2020). Osteocyte RANKL is required for cortical bone loss with age and is induced by senescence. JCI Insight *5*, 138815. https://doi.org/10.1172/jci.insight.138815.

Lim, S., and Kaldis, P. (2013). Cdks, cyclins and CKIs: roles beyond cell cycle regulation. Development *140*, 3079–3093. https://doi.org/10.1242/dev.091744.

Logan, M., Martin, J.F., Nagy, A., Lobe, C., Olson, E.N., and Tabin, C.J. (2002). Expression of Cre Recombinase in the developing mouse limb bud driven by a Prxl enhancer. Genesis *33*, 77–80. https://doi.org/10.1002/gene.10092.

Malumbres, M. (2014). Cyclin-dependent kinases. Genome Biol. *15*, 122. https://doi.org/10.1186/gb4184.

Manolagas, S.C., and Parfitt, A.M. (2010). What old means to bone. Trends Endocrinol. Metabol. *21*, 369–374. https://doi.org/10.1016/j.tem.2010.01.010.

Martinez-Fabregas, J., Wang, L., Pohler, E., Cozzani, A., Wilmes, S., Kazemian, M., Mitra, S., and Moraga, I. (2020). CDK8 fine-tunes IL-6 transcriptional activities by limiting STAT3 resident time at the gene Loci. Cell Rep. *33*, 108545. https://doi.org/10.1016/j.celrep.2020.108545.

Mbalaviele, G., Jaiswal, N., Meng, A., Cheng, L., Van Den Bos, C., and Thiede, M. (1999). Human mesenchymal stem cells promote human osteoclast differentiation from CD34+ bone marrow hematopoietic progenitors. Endocrinology *140*, 3736–3743. https://doi.org/10.1210/endo.140.8.6880.

McCleland, M.L., Soukup, T.M., Liu, S.D., Esensten, J.H., de Sousa e Melo, F., Yaylaoglu, M., Warming, S., Roose-Girma, M., and Firestein, R. (2015). *Cdk8* deletion in the Apc^{Min} murine tumour model represses EZH2 activity and accelerates tumourigenesis. J. Pathol. *237*, 508–519. https://doi.org/10.1002/path.4596.

Menzl, I., Zhang, T., Berger-Becvar, A., Grausenburger, R., Heller, G., Prchal-Murphy, M., Edlinger, L., Knab, V.M., Uras, I.Z., Grundschober, E., et al. (2019). A kinase-independent role for CDK8 in BCR-ABL1(+) leukemia. Nat. Commun. *10*, 4741. https://doi.org/10.1038/s41467-019-12656-x.

Ozaki, K., Yamada, T., Horie, T., Ishizaki, A., Hiraiwa, M., Iezaki, T., Park, G., Fukasawa, K., Kamada, H., Tokumura, K., et al. (2019). The L-type amino acid transporter LAT1 inhibits osteoclastogenesis and maintains bone homeostasis through the mTORC1 pathway. Sci. Signal. *12*, eaaw3921. https://doi.org/10.1126/scisignal.aaw3921.

Piemontese, M., Almeida, M., Robling, A.G., Kim, H.N., Xiong, J., Thostenson, J.D., Weinstein, R.S., Manolagas, S.C., O'Brien, C.A., and Jilka, R.L. (2017). Old age causes de novo intracortical bone remodeling and porosity in mice. JCI Insight *2*, 93771. https://doi.org/10.1172/jci.insight.93771.

Ponnapakkam, T., Katikaneni, R., Sakon, J., Stratford, R., and Gensure, R.C. (2014). Treating osteoporosis by targeting parathyroid hormone to bone. Drug Discov. Today *19*, 204–208. https://doi.org/10.1016/j.drudis.2013.07.015.

Rachner, T.D., Khosla, S., and Hofbauer, L.C. (2011). Osteoporosis: now and the future. Lancet *377*, 1276–1287. https://doi.org/10.1016/S0140-6736(10)62349-5.

Reid, I.R. (2015). Efficacy, effectiveness and side effects of medications used to prevent fractures. J. Intern. Med. *277*, 690–706. https://doi.org/10.1111/joim.12339.

Rodda, S.J., and McMahon, A.P. (2006). Distinct roles for Hedgehog and canonical Wnt signaling in specification, differentiation and maintenance of osteoblast progenitors. Development *133*, 3231–3244. https://doi.org/10.1242/dev.02480.

Shen, G., Ren, H., Shang, Q., Zhao, W., Zhang, Z., Yu, X., Tang, K., Tang, J., Yang, Z., Liang, D., and Jiang, X. (2020). Foxf1 knockdown promotes BMSC osteogenesis in part by activating the Wnt/β-catenin signalling pathway and prevents ovariectomy-induced bone loss. EBioMedicine *52*, 102626. https://doi.org/10.1016/j.ebiom.2020.102626.

Sundaram, K., Senn, J., Yuvaraj, S., Rao, D.S., and Reddy, S.V. (2009). FGF-2 stimulation of RANK ligand expression in Paget's disease of bone. Mol. Endocrinol. *23*, 1445–1454. https://doi.org/10.1210/me.2009-0078.

Teitelbaum, S.L., and Ross, F.P. (2003). Genetic regulation of osteoclast development and function. Nat. Rev. Genet. *4*, 638–649. https://doi.org/10.1038/nrg1122.

Tokuzawa, Y., Yagi, K., Yamashita, Y., Nakachi, Y., Nikaido, I., Bono, H., Ninomiya, Y., Kanesaki-Yatsuka, Y., Akita, M., Motegi, H., et al. (2010). Id4, a new candidate gene for senile osteoporosis, acts as a molecular switch promoting osteoblast differentiation. PLoS Genet. *6*, e1001019. https://doi.org/10.1371/journal.pgen.1001019.

Westerling, T., Kuuluvainen, E., and Mäkelä, T.P. (2007). Cdk8 is essential for preimplantation mouse development. Mol. Cell Biol. *27*, 6177–6182. https://doi.org/10.1128/mcb.01302-06.

Xu, R., Shen, X., Si, Y., Fu, Y., Zhu, W., Xiao, T., Fu, Z., Zhang, P., Cheng, J., and Jiang, H. (2018). MicroRNA-31a-5p from aging BMSCs links bone formation and resorption in the aged bone marrow microenvironment. Aging Cell *17*, e12794. https://doi.org/10.1111/acel.12794.

Yamamoto, T., Hinoi, E., Fujita, H., Iezaki, T., Takahata, Y., Takamori, M., and Yoneda, Y. (2012). The natural polyamines spermidine and spermine prevent bone loss through preferential disruption of osteoclastic activation in ovariectomized mice. Br. J. Pharmacol. *166*, 1084–1096. https://doi.org/10.1111/j.1476-5381.2012.01856.x.

You, L., Pan, L., Chen, L., Gu, W., and Chen, J. (2016). MiR-27a is essential for the shift from osteogenic differentiation to adipogenic differentiation of mesenchymal stem cells in postmenopausal osteoporosis. Cell. Physiol. Biochem. *39*, 253–265. https://doi.org/10.1159/000445621.

Yu, B., Huo, L., Liu, Y., Deng, P., Szymanski, J., Li, J., Luo, X., Hong, C., Lin, J., and Wang, C.Y. (2018). PGC-1α controls skeletal stem cell fate and bone-fat balance in osteoporosis and skeletal aging by inducing TAZ. Cell Stem Cell *23*, 193–209.e5. https://doi.org/10.1016/j.stem.2018.06.009.

Zhong, L., Yao, L., Tower, R.J., Wei, Y., Miao, Z., Park, J., Shrestha, R., Wang, L., Yu, W., Holdreith, N., et al. (2020). Single cell transcriptomics identifies a unique adipose lineage cell population that regulates bone marrow environment. Elife *9*, e54695. https://doi.org/10.7554/eLife.54695.

Zhou, B.O., Yue, R., Murphy, M.M., Peyer, J.G., and Morrison, S.J. (2014). Leptin-receptor-expressing mesenchymal stromal cells represent the main source of bone formed by adult bone marrow. Cell Stem Cell *15*, 154–168. https://doi.org/10.1016/j.stem.2014.06.008.

Zhou, S., Greenberger, J.S., Epperly, M.W., Goff, J.P., Adler, C., Leboff, M.S., and Glowacki, J. (2008). Age-related intrinsic changes in human bone-marrow-derived mesenchymal stem cells and their differentiation to osteoblasts. Aging Cell *7*, 335–343. https://doi.org/10.1111/j.1474-9726.2008.00377.x.

Stem Cell Reports, Volume *17*

Supplemental Information

The role of CDK8 in mesenchymal stem cells in controlling osteoclastogenesis and bone homeostasis

Takanori Yamada, Kazuya Fukasawa, Tetsuhiro Horie, Takuya Kadota, Jiajun Lyu, Kazuya Tokumura, Shinsuke Ochiai, Sayuki Iwahashi, Akane Suzuki, Gyujin Park, Rie Ueda, Megumi Yamamoto, Tatsuya Kitao, Hiroaki Shirahase, Hiroki Ochi, Shingo Sato, Takashi Iezaki, and Eiichi Hinoi

Supplementary Table 1. List of primers used for genotyping

Gene	Forward (5’-3’)	Reverse (5’-3’)
Cre	GAACCTGATGGACATGTTCAGG	AGTGCGTTCGAACGCTAGAGCCTGT
CDK8	CGTAGGTAGCAATCTGGTCGGGGT	CAGGTACACAGGCTGGATTTGCAC

Supplementary Table 2. List of primers used for deletion PCR.

Gene	Forward (5’-3’)	Reverse (5’-3’)
CDK8	CTTCCCTCTTCCCAGAGGAC	CAACCCCTTTTGAGGTTGAA

Supplementary Table 3. List of primers used for real-time PCR.

Gene	Forward (5’-3’)	Reverse (5’-3’)
Acp5	AGCTTCTCTGCCCTGGTACTC	TGGCTGTGGGATCAGTTGGTG
Cdkn1a	TCGCTGTCTTGCACTCTGGTGT	CCAATCTGCGCTTGGAGTGATAG
Ctsk	CAACTCTACTTCCCTTCTCTCTGC	GAGGACTCCAATGTCTACCAGC
Gapdh	AGGTCGGTGTGAACGGATTTG	TGTAGACCATGTAGTTGAGGTCA
Icam1	TTCACACTGAATGCCAGCTC	GTCTGCTGAGACCCCTCTTG
Ifi204	GGAAAGAGACAACCAAGAGC	TGGCTTGTAGTTGATGTAGG
Irf1	TCCAAGTCCAGCCGAGACACTA	ACTGCTGTGGTCATCAGGTAGG
Irf8	TCCAAGTCCAGCCGAGACAC	ACTGCTGTGGTCATCAGGTA
Mmp9	TGTGCGTTATGGTTCAGGTCAG	CTGCCAGGAAGACACTTGGTTATC
Smad7	AAGATCGGCTGTGGCATC	CCAACAGCGTCCTGGAGT
Spi1	ATCCAGAAGGGCAACCGCAAG	TCTTGACTTTCTTCACCTCGCCTG
Tap1	GACTCCTTGCTCTCCACTCA	AACGCTGTCACCGTTCCAGG
Tnfrsf11b	CTGCAATACACACACTCATCACT	ACCCAGAAACTGGTCATCAGC
Tnfsf11	TGTACTTTCGAGCGCAGATG	CCACAATGTGTTGCAGTTCC

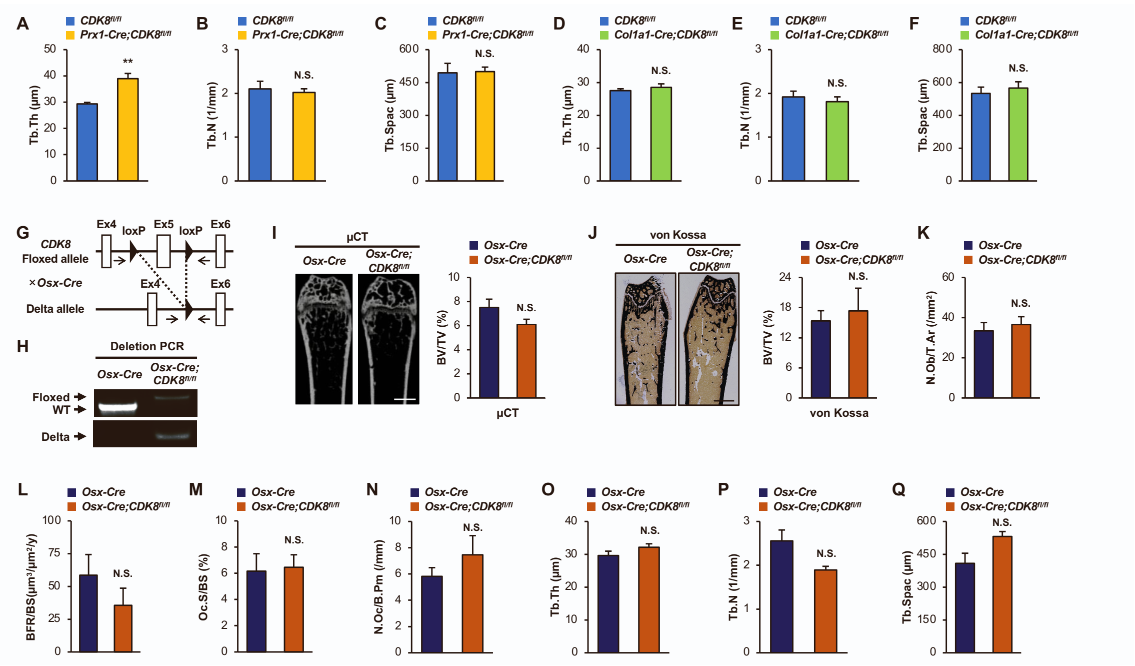

Supplementary Figure 1. Skeletal phenotype analysis of conditional MSC-specific, osteoblast-specific, and osteoprogenitor-specific *CDK8* knockout mice.

(A-C) Tb.Th (A), Tb.N (B) and Tb.Spac (C) measured by μCT of femurs from *CDK8*$^{fl/fl}$ and *Prx1-Cre;CDK8*$^{fl/fl}$ mice (n = 6-7, $^{**}P < 0.01$). (D-F) Tb.Th (D), Tb.N (E) and Tb.Spac (F) measured by μCT of femurs from *CDK8*$^{fl/fl}$ and *Col1a1-Cre;CDK8*$^{fl/fl}$ mice (n = 6-7). (G) Schematic diagram of generation of tissue-specific *CDK8* knockout mice. Black arrows represent primer binding sites. (H) Deletion efficiency of *CDK8* in the marrow-flushed bone of *Osx-Cre;CDK8*$^{fl/fl}$ mice at the genomic DNA level. (I) μCT analysis and BV/TV measurement of femurs from *Osx-Cre* and *Osx-Cre;CDK8*$^{fl/fl}$ mice (n = 4-5). (J to N) BV/TV determined by von Kossa staining (J), N.Ob/T.Ar (K), BFR/BS (L), Oc.S/BS (M) and N.Oc/B.Pm (N) of femur from *Osx-Cre* and *Osx-Cre;CDK8*$^{fl/fl}$ mice (n = 4-5). (O-Q) Tb.Th (O), Tb.N (P) and Tb.Spac (Q) measured by μCT of femurs from *CDK8*$^{fl/fl}$ and *Osx-Cre;CDK8*$^{fl/fl}$ mice (n = 4-5). All mice used in this study were female. Scale bars, 1 mm (I and J). μCT, micro-computed tomography; Tb.Th, Trabecular thickness; Tb.N, Trabecular number; Tb.Spac; Trabecular spacing; BV/TV, bone volume/tissue volume; N.Ob/T.Ar, number of osteoblasts/tissue area; BFR/BS, bone formation rate/bone surface; Oc.S/BS, osteoclast surface/bone surface; N.Oc/B.Pm, number of osteoclasts/bone perimeter; N.S., not significant.

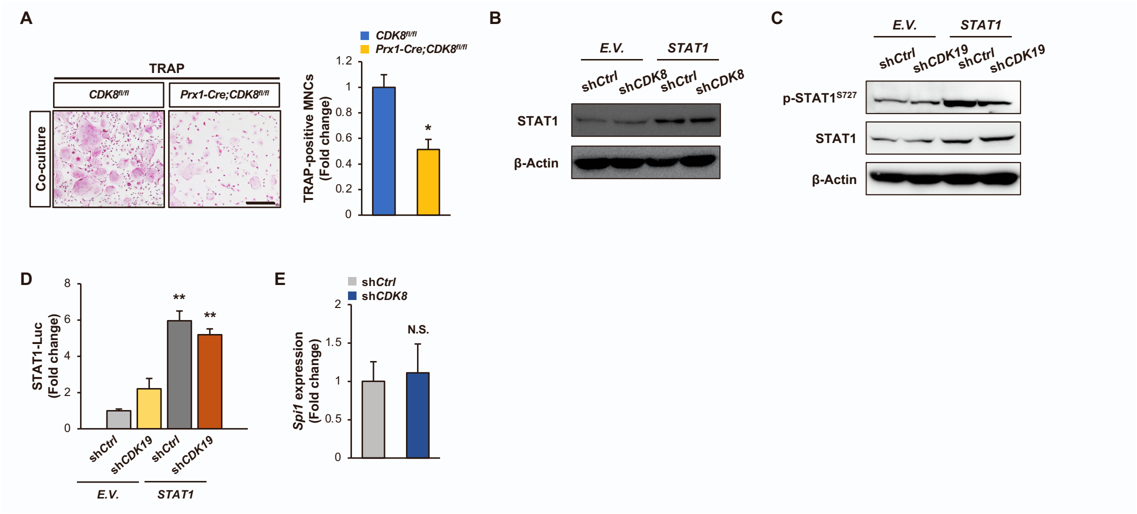

Supplementary Figure 2. Co-culture of MSCs from *Prx1-Cre;CDK8$^{fl/fl}$* mice and WT-BMMs, and expression profiles of *CDK8* and *CDK19* knockdown MSCs.

(A) BMMs prepared from WT mice were co-cultured with MSCs from *Prx1-Cre;CDK8$^{fl/fl}$* mice, followed by TRAP staining (n = 3 independent replicates, $^{*}P < 0.05$). (B) Protein levels of STAT1 in MSCs infected with sh*CDK8* in combination with *STAT1* expression vector. β-Actin served as a loading control. (C) Protein levels of STAT1 and p-STAT1^{Ser727} in MSCs infected with sh*CDK19* in combination with *STAT1* expression vector. β-Actin served as a loading control. (D) GAS-luc activity in MSCs infected with sh*CDK19* in combination with *STAT1* expression vector (n = 3 independent replicates, $^{**}P < 0.01$). (E) mRNA levels of *Spi1* in MSCs infected with sh*CDK8* (n = 4 independent replicate). Scale bar, 800 µm (A). BMM, bone marrow macrophage; MSC, mesenchymal stem cell; TRAP, tartrate-resistant acid phosphatase; sh*Ctrl*, sh*Control*; E.V., empty vector; N.S., not significant.

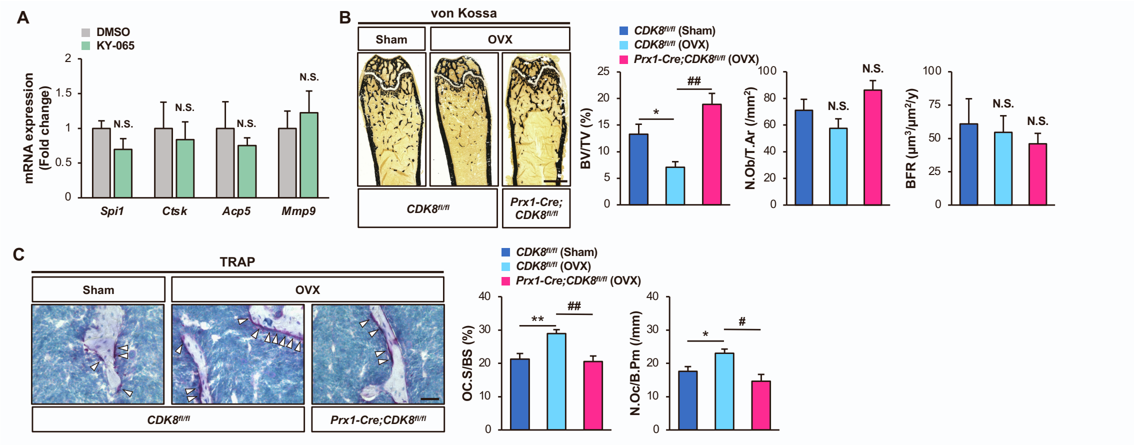

Supplementary Figure 3. Expression levels of osteoclastic marker genes in BMMs treated by KY-065, and the protective effect of MSC-specific *CDK8* deficiency on ovariectomy-induced bone loss.

(A) mRNA levels of *Spi1*, *Ctsk*, *Acp5* and *Mmp9* in MSCs treated with 30 nM KY-065 (n = 3 independent replicates). (B) BV/TV measured by von Kossa staining, N.Ob/T. Ar determined by toluidine blue staining and BFR/BS determined by calcein labeling of femurs from $CDK8^{fl/fl}$ and $Prx1\text{-}Cre;CDK8^{fl/fl}$ mice (n = 5-8, $^{*}P < 0.05$ ($CDK8^{fl/fl}$ (sham) vs $CDK8^{fl/fl}$ (OVX)), $^{\#\#}P < 0.01$ ($CDK8^{fl/fl}$ (OVX) vs $Prx1\text{-}Cre;CDK8^{fl/fl}$(OVX))). (C) Oc.S/BS and N.Oc/B.Pm determined by TRAP staining of femur (n = 5-8, $^{*}P < 0.05$ ($CDK8^{fl/fl}$ (sham) vs $CDK8^{fl/fl}$ (OVX)), $^{**}P < 0.01$ ($CDK8^{fl/fl}$ (sham) vs $CDK8^{fl/fl}$ (OVX)), $^{\#}P < 0.05$ ($CDK8^{fl/fl}$ (OVX) vs $Prx1\text{-}Cre;CDK8^{fl/fl}$(OVX)), $^{\#\#}P < 0.01$ ($CDK8^{fl/fl}$ (OVX) vs $Prx1\text{-}Cre;CDK8^{fl/fl}$(OVX))). Scale bars, 1 mm (B), 50 μm (C). OVX, ovariectomy; BV/TV, bone volume/tissue volume; N.Ob/T.Ar, number of osteoblasts/tissue area; BFR/BS, bone formation rate/bone surface; TRAP, tartrate-resistant acid phosphatase; Oc.S/BS, osteoclast surface/bone surface; N.Oc/B.Pm, number of osteoclasts/bone perimeter; N.S., not significant.
